# Adhesion receptor ADGRG2/GPR64 is in the GI-tract selectively expressed in mature intestinal tuft cells

**DOI:** 10.1016/j.molmet.2021.101231

**Published:** 2021-04-05

**Authors:** Kaare V. Grunddal, Sarah Tonack, Kristoffer L. Egerod, Jonathan James Thompson, Natalia Petersen, Maja S. Engelstoft, Constance Vagne, Céline Keime, Gérard Gradwohl, Stefan Offermanns, Thue W. Schwartz

**Affiliations:** 1 NNF Center for Basic Metabolic Research, Faculty of Health and Medical Sciences, University of Copenhagen, Blegdamsvej 3, 2200 Copenhagen, Denmark; 2 Department of Biomedical Sciences, Faculty of Health and Medical Sciences, University of Copenhagen, Copenhagen, Denmark; 3 Department of Pharmacology, Max-Planck-Institute for Heart and Lung Research, Ludwigstrasse 43, 61231 Bad Nauheim, Germany; 4 Department of Development and Stem Cells, Institut de Génétique et de Biologie Moléculaire et Cellulaire (IGBMC), Illkirch, France; 5 Centre National de la Recherche Scientifique, UMR7104, Illkirch, France; 6 Institut National de la Santé et de la Recherche Médicale, U1258, Illkirch, France; 7 Université de Strasbourg, Illkirch, France; 8 Plateforme GenomEast, Infrastructure France Génomique, Illkirch, France

**Keywords:** ADGRG2, GPR64, GPCRs, Tuft cells, Chemosensory cells, AC-TUB, acetylated α-tubulin, ADGRG2, adhesion G protein-coupled receptor G2, CK18, cytokeratin 18, DCLK1, doublecortin like kinase 1, DRD3, dopamine receptor D3, GI, gastrointestinal, GLP-1, glucagon-like peptide-1, GPCR, G protein-coupled receptor, GPRC5C, G protein-coupled receptor family C group 5 member C, GPR64, G protein-coupled receptor 64, HPGDS, hematopoietic prostaglandin D synthase, IUPHAR, International Union of Basic and Clinical Pharmacology, MOE, main olfactory epithelium, ONS, olfactory sensory neurons, PGP9.5, protein gene product 9.5, PTGS1, prostaglandin-endoperoxide Synthase 1, PTGS2, prostaglandin-endoperoxide Synthase 2, SUCNR1, succinate Receptor 1, TMX, tamoxifen, TRC, taste-receptor cells, TRPM5, transient receptor potential cation channel, subfamily M, member 5

## Abstract

**Objective:**

GPR64/ADGRG2 is an orphan Adhesion G protein-coupled receptor (ADGR) known to be mainly expressed in the parathyroid gland and epididymis. This investigation aimed to delineate the cellular expression of GPR64 throughout the body with focus on the gastrointestinal (GI) tract.

**Methods:**

Transgenic *Gpr64*^*mCherry*^ reporter mice were histologically examined throughout the body and reporter protein expression in intestinal tuft cells was confirmed by specific cell ablation. The GPCR repertoire of intestinal *Gpr64*^*mCherry*^-positive tuft cells was analyzed by quantitative RT-PCR analysis and *in situ* hybridization. The *Gpr64*^*mCherry*^ was crossed into the general tuft cell reporter *Trpm5*^*GFP*^ to generate small intestinal organoids for time-lapse imaging. Intestinal tuft cells were isolated from small intestine, FACS-purified and transcriptionally compared using RNA-seq analysis.

**Results:**

Expression of the *Gpr64*^*mCherry*^ reporter was identified in multiple organs and specifically in olfactory microvillous cells, enteric nerves, and importantly in respiratory and GI tuft cells. In the small intestine, cell ablation targeting Gpr64-expressing epithelial cells eliminated tuft cells. Transcriptional analysis of small intestinal *Gpr64*^*mCherry*^ -positive tuft cells confirmed expression of Gpr64 and the chemo-sensors Sucnr1, Gprc5c, Drd3, and Gpr41/Ffar3. Time-lapse studies of organoids from *Trpm5*^*GFP*^*:Gpr64*^*mCherry*^ mice revealed sequential expression of initially *Trpm5*^*GFP*^ and subsequently also *Gpr64*^*mCherry*^ in maturing intestinal tuft cells. RNA-seq analysis of small intestinal tuft cells based on these two markers demonstrated a dynamic change in expression of transcription factors and GPCRs from young to mature tuft cells.

**Conclusions:**

GPR64 is expressed in chemosensory epithelial cells across a broad range of tissues; however, in the GI tract, GPR64 is remarkably selectively expressed in mature versus young immunoregulatory tuft cells.

## Highlights

• GPR64-RFP is expressed mainly in chemosensory epithelial cells, including tuft cells.
• Maturing intestinal tuft cells initially express Trpm5 and subsequently Gpr64.
• Mature intestinal Gpr64+ tuft cells express Sucnr1, Gprc5c, Drd3, and Gpr41/Ffar3.
• RNAseq analysis reveals dynamic transcriptional change of mature vs. young tuft cells.

## 1 Introduction

In the gastrointestinal (GI) tract, the composition of ingested food and gut microbiota metabolites are monitored by specialized chemosensory cells scattered throughout the GI epithelium modulating physiological functions in response to conditions in the lumen. These epithelial chemosensory cells include enteroendocrine cells as well as tuft cells which are all continuously generated from stem cells located in the crypts of the mucosa. Like the remaining intestinal epithelium, these sensory cells migrate up the villus and are extruded at the tip [1]. Enteroendocrine cells sense nutrient and microbial metabolites through a number of different G protein-coupled receptors (GPCRs) and in response secrete hormones regulating both gut physiology and whole body metabolism [2]. The more poorly characterized intestinal tuft cells are currently emerging as important immunomodulatory sensors of parasites.

Tuft cells (or brush cells) were identified over 60 years ago through their distinct ultrastructural features: a large, blunt apical brush-like process and a well-developed apical tubulovesicular system [3]. Since then, tuft cells have been identified in multiple body cavities including the GI and respiratory tract, specifically in the tracheal epithelium [3,4], alveolar lining and bronchioles [5], the gastric ventricle [[6], [7], [8], [9]], small and large intestine [[10], [11], [12], [13]] as well as pancreatic duct [8,12]. Based on their morphological resemblance with lingual taste-receptor cells, intestinal tuft cells were early on suspected to be chemosensory cells [14]. This notion is further supported by their expression of taste-related GPCRs [15] as well as many signaling proteins involved in the taste transduction pathway, including TRPM5 [16] and α-gustducin [17]. Recently, key publications have identified mouse intestinal tuft cells as critical sensory sentinels mediating the host defense against parasitic protozoa and helminths. Intestinal tuft cells respond to parasitic infection by increasing their secretion of IL25, which in turn promotes proliferation of the lamina propria type 2 innate lymphoid cells (ILC2) which secrete various cytokines, including IL13. Interestingly, these cytokines promote tuft- and goblet cell hyperplasia in a feed forward loop known as the tuft-ILC2 circuit ultimately resulting in parasitic clearance [13,18,19]. How intestinal tuft cells sense their microenvironment and potential pathogens is, however, less clear.

Recent studies point to key metabolites as triggers of tuft cell-driven type 2 immunity. Thus, tuft cells of the GI tract have been shown to be highly enriched for certain metabolite GPCRs such as the succinate receptor Sucnr1/Gpr91 and the short chain fatty acid (SCFA) receptor Ffar3/Gpr41 [6,16]. Both receptors respond to metabolites produced by luminal microbes and are therefore candidate receptors for pathogen recognition. Both the helminth *Nippostrongylus brasiliensis* and a tritrichomonad protist secrete succinate, but interestingly, *in vivo* sensing of the tritrichomonand, but not *N. brasiliensis,* requires Sucnr1 to trigger the tuft-ILC2 circuit [20,21]. This suggests the existence of other mechanisms for sensing of helminths. Thus, metabolite GPCRs are emerging as potentially important part of tuft cell function.

Molecular markers restricted to tuft cell recognition were until recently rare, as many are shared with other cell types. Besides the taste transduction-related proteins such as α-gustducin and TRPM5, many applied markers relate to the tuft cells’ unique cytoskeletal features like cytokeratin 18 (CK18) filaments highlighting the perinuclear region and cell periphery [9,12,22], acetylated tubulin (Ac-tub) [23] and doublecortin-like kinase 1 protein (DCLK1, also called DCAMKL-1) [13,18,19,24,25] highlighting the dense apical microtubule network. Intestinal tuft cells have also been shown to express the rate limiting enzymes of prostanoid biosynthesis prostaglandin-endoperoxide synthase 1 and 2 (PTGS1 and PTGS2) and hematopoietic prostaglandin-D synthase (HPGDS) producing prostaglandin-D2 [16,25]. More recently, transcription factors associated with intestinal tuft cell differentiation, GFI1B [13,18,19,26], POU2F3 and SOX9 [13], and secretory product IL-25 [18,21] have been used in the identification of intestinal tuft cells. Unambiguous identification of tuft cells, however, still requires a combination of multiple molecular markers [27].

Gpr64 (Adgrg2 or He6) belongs to the subfamily G (also known as Group VIII) of the family of adhesion GPCRs (ADGRs) together with Gpr56 (Adgrg1), Gpr97 (Adgrg3), Gpr112 (Adgrg4), Gpr114 (Adgrg5), Gpr126 (Adgrg6), and Gpr128 (Adgrg7) [28]. Like other ADGRs, Gpr64 is characterized by a 7 transmembrane (7TM) domain preceded by a very large N terminus, which contains a GPCR Autoproteolysis Inducing (GAIN) domain with a canonical GPCR Proteolytic Site (GPS) subdomain, which is autoproteolytically cleaved during receptor synthesis. However, the extracellular N-Terminal Fragment (NTF) and the 7TM domain remains associated at the cell membrane as the N-terminal end of the 7TM-domain constitutes the last beta-strand of the coiled beta-sheets of the GPS subdomain [[29], [30], [31]]. In ADGRs, the dogma is that when the NTF interacts with its ligand and dissociates, the suppression of the 7TM domain is relieved allowing for intracellular signaling via multiple intracellular G-protein pathways such as Gs, Gα_12/13_, Gα_q_ [31] and Gi [30]. The ECD of GPR64 is predicted to be strongly O-glycosylated and to function like a mucin-like domain [32,33]. In contrast to most ADGRs, full length GPR64 is surprisingly signaling with high constitutive activity through Gq and G12/13 [31], while the truncated 7TM domain of GPR64 signals strongly through Gs and cAMP, and has been shown to interact with the Ca^2+^-sensing receptor (CaSR) in the parathyroid [34].

In healthy human and mouse tissue, Gpr64 expression was initially identified in the epididymis, where the receptor was first discovered [32] and subsequently in the parathyroid gland [34]. Male Gpr64-knockout mice display fluid dysregulation and spermatozoa obstruction in the efferent ducts of the epididymis resulting in infertility, with no other apparent tissue deformation [35]. Initial genetic studies on human Gpr64 confirmed a crucial role of GPR64 in human male fertility [36]. Gpr64 has also been shown to be upregulated in various carcinomas including kidney, prostate, lung, and breast cancer, as well as Ewing sarcomas [37], where it appears to promote adhesion and migration, but not proliferation [31,37].

In this study, we characterized a novel transgenic *Gpr64*^*mCherry*^ reporter mouse, which expresses the fluorescent protein mCherry under the control of the Gpr64/Adgrg2 promoter. We present a novel expression profile of Gpr64 in numerous different tissues including olfactory sensory neurons and; importantly, tuft cells of the GI tract and respiratory system. The repertoire of GPCRs expressed by small intestinal *Gpr64*^*mCherry*^-positive tuft cells was shown to include a number of receptors for microbial metabolites. Importantly, a dual *Trpm5*^*GFP*^*:Gpr64*^*mCherry*^ reporter mouse revealed that Gpr64 was preferentially expressed on mature tuft cells of the villi and enable RNA-seq analysis of the transcriptional fingerprint of young versus mature tuft cells.

## 2 Material and methods

### 2.1 Compounds

Recombinant murine IL-4 (214-14) and recombinant murine IL-13 (210-13) were purchased from PeproTech.

### 2.2 Animals

To generate transgenic mice expressing mCherry under the control of the Adgrg2 (Gpr64) promoter (*Gpr64*^*mCherry*^) (Figs. S1A and B), we used the BAC clone RP23-232E24 (CHORI, CA, USA) from mouse X chromosome containing the Gpr64 gene. The partial coding sequence of the Gpr64 gene, including exon 3 (containing the ATG start codon) to exon 8, on the BAC was replaced by a cassette carrying the mCherry cDNA followed by a polyadenylation signal and an FRT-flanked ampicillin resistance cassette (β-lactamase) using the Red/ET recombination kit (Gene Bridges, Heidelberg, Germany). After fragment length polymorphism-mediated verification and excision of the ampicillin gene, transgenic founder lines were generated via pronucleus injection into CD-1 oocytes. At least two different founders were used to generate Gpr64 reporter lines, which all showed consistent expression patterns for mCherry. Animals were backcrossed on a C57BL/6 background.

For the generation of tamoxifen-inducible, villin-specific, Gpr64-dependent, cell-ablation mice (*Vil-CreERT2;GPR64*^*DTA*^), transgenic GPR64-eGFP-DTA (*Gpr64*^*DTA*^) mice were generated (Figs. S1C and D) and crossed with Villin-CreERT2 (*Vil-CreERT2)* mice [38]. Mice were maintained on a C57BL/6 J background and genetically matched Cre-negative *Gpr64*^*DTA*^ mice were used as controls. For induction of Cre-mediated recombination, mice were treated on five consecutive days with 1 mg tamoxifen intraperitoneally. At different timepoints after induction, the mice were sacrificed by CO_2_ and the intestine dissected into parts for RNA and histological analysis.

The *Trpm5*^*GFP*^ reporter mice (kindly provided by Dr. Robert Margolskee, Monell Chemical Senses Center, Philadelphia) contain a Trpm5-GFP construct including 11 kb of mouse TrpM5 5′ flanking sequence, TrpM5 exon 1 (untranslated), intron 1, and the untranslated part of exon 2, and GFP [39]. Double transgenic *Trpm5*^*GFP*^:*Gpr64*^*mCherry*^ reporter mice were generated by crossing *Trpm5*^*GFP*^ mice with *Gpr64*^*mCherry*^ mice. Wild-type C57BL6/J mice tissue was used as negative fluorescence control for all reporter strains. Male mice were used in *Gpr64*^*mCherry*^ studies, while both female and male mice were used in *Trpm5*^*GFP*^:*Gpr64*^*mCherry*^ studies.

All mice were housed in a temperature- and humidity-controlled environment under a 12-h light/dark phase cycle with ad libitum access to water and chow diet under pathogen-free conditions. All experiments were approved by the Danish Animal Inspectorate conducted in accordance with institutional guidelines and the Institutional Animal Care and Use Committee of the Regierungspräsidium Darmstadt and in accord with Directive 2010/63/EU of the European Parliament on the protection of animals used for scientific purposes.

### 2.3 Immunohistochemistry

Twelve-week-old male transgenic *Gpr64*^*mCherry*^ mice were euthanized by cervical dislocation and tissues were excised, rinsed in phosphate buffered saline (PBS), fixed in freshly-made 4% paraformaldehyde PBS for 24 h at 4 °C, cryoprotected for 24 h (20% sucrose PBS) at 4 °C and embedded in mounting medium (361603 E, VWR chemicals, Soeborg, Denmark) for cryotomy, plunge-frozen in dry ice-cooled isopentane and subsequently stored at −80 °C. Sections (8 μm) were cut using a cryostat (CM3050 S, Leica, Wetzlar, Germany), air-dried for 30 min at room temperature, and either washed in PBS and boiled in 0.01 M citrate buffer (pH 6.0) for 15 min and allowed to cool for 30 min, or washed in PBS. Section were then incubated with blocking buffer (2% bovine serum albumin) for 10 min at room temperature, before being incubated with primary antibodies (Table S1) overnight at 4 °C. Sections were washed, incubated with fluorophore-conjugated secondary antibodies (Table S1) for 1 h. Finally, coverslips were mounted with ProLong Gold Antifade Mountant with DAPI (P-36931, Thermofisher). Sections were analyzed using an IX71 Olympus microscope and XM10 Olympus camera. Pseudo-color application and picture merging was performed in Adobe Photoshop. Control studies in the regions of interest revealed no unspecific labeling of the secondary antibodies.

### 2.4 Whole mount preparation of the intestinal plexus

A section of 2 cm of the duodenum and ileum was collected and stored in ice cold PBS. The mucosa was gently removed and the submucosal plexus detached from the mucosa by sharp microdissection. The submucosal plexus was removed, fixed for 4 h in 4% ice cold paraformaldehyde and washed 3 times for 10 min with PBS at room temperature. The circular smooth muscle layer was carefully removed with forceps, leaving the myenteric plexus on top of the longitudinal smooth muscle layer. This preparation was fixed accordingly. The immunofluorescence staining was performed on free floating sections in 2 mL Eppendorf tubes. After 1 h incubation in blocking buffer, primary antibodies (Table S1) were incubated at 4 °C overnight. The sections were washed 3 times 10 min with PBS and incubated in the secondary antibodies (Table S1). After 3 times 10 min washes with PBS the sections were embedded on a slide and covered with Fluoromount W. Pictures were taken using a Leica TCS SP5 microscope (Leica).

### 2.5 Intestinal organoid culture

Intestinal organoids were generated from 12 to 16-week-old double transgenic *Trpm5*^*GFP*^:*Gpr64*^*mCherry*^ reporter mice, which both labels intestinal tuft cells. In brief, non-fasted mice were sacrificed by cervical dislocation, the duodenum was excised, and crypts were released by incubation in PBS containing 2 mM EDTA for 1 h at 4 °C and seeded in 24-well plates in Matrigel (BD Biosciences), where they grew into organoids as previously described (Sato 2009). For maintenance, organoids were split every 4–6 days. Organoids were cultured in small intestinal growth medium: advanced Dulbecco's modified Eagle's medium (DMEM)/F12 containing 2% penicillin-streptomycin, 10 mmol L^−1^ HEPES, 1 μmol L^−1^ N-acetylcysteine; Glutamax and B27 (from Invitrogen, according to manufacturer's instructions, 50 ng mL^−1^ EGF, 500 ng mL^−1^ R-spondin 1, 100 ng mL^−1^ Noggin). To promote expansion of the tuft cell population for time-lapse imaging, we included 400 ng mL^−1^ recombinant murine IL-4 and with 400 ng mL^−1^ recombinant murine IL-13 was added to the growth medium [13].

### 2.6 Time-lapse imaging

*Trpm5*^*GFP*^:*Gpr64*^*mCherry*^ organoids were seeded into matrigel in flat-bottom, chambered coverglass (Cat no. 155411, Thermo Scientific) and cultured in regular small intestinal growth medium with the recombinant murine IL-4 and Il-13. mCherry, GFP and brightfield images were captured every 3 h during up to 60-h period using a wide-field Nikon Ti-E microscope equipped with a humidity controlled, thermostatic chamber with 5% CO2 air influx. Time-lapse experiments were started 4 h after splitting and platting.

### 2.7 In situ hybridization

Twelve-to 18-week-old male transgenic *Gpr64*^*mCherry*^ mice were euthanized by cervical dislocation and jejunum was excised, rinsed with cold PBS, and transferred to freshly-made 4% paraformaldehyde for 24-h fixation at room temperature. Tissue was then stored in 70% alcohol before infiltration (Shandon Excelsior; Thermo Fisher) and embedding in paraffin blocks. 5-μm sections were cut using a microtome (RM2125; Leica) and mounted onto Superfrost Plus Slides (Thermo Scientific) at 60 °C for 1 h.

The distribution of Gpr64, Sucnr1, Glp1r, Drd3 Gprc5c, and Ffar3 mRNA in murine jejunum was investigated using the RNAscope 2.0- or 2.5HD (Cat. no. 320487 and cat. no. 322350) Detection kit (Red) assays and probes (Table S2) purchased from Advanced Cell Diagnostics. In brief, sections were dewaxed in xylene and alcohol and allowed to airdry before incubation with pretreatment 1 solution for 10 min at room temperature, boiled in pretreatment 2 solution for 14 min (mouse sections) or 15 min (human sections) and protease digested in pretreatment 3 solution at 40 °C for 30 min. Slides were then incubated with probe-solutions at 40 °C for 2 h and subsequently treated according to RNAscope 2.0- or 2.5HD Detection kit (Red) assay user manual. Finally, mRNA was stained with Fast Red dye and sections were immunofluorescently labeled as described below.

### 2.8 FACS purification of intestinal Gpr64^mCherry^ cells

Five male 12–16-week-old *Gpr64*^*mCherry*^ transgenic mice were euthanized and duodenum and jejunum was excised, inverted, inflated and digested for 20 min with 0.13 Wünsch units of Liberase (Roche, Indianapolis, IN) in Dulbecco's Modified Eagle Medium (DMEM, low glucose) 1885 while being slowly shaken in a water bath at 37 °C. Every fifth minute the tissue was vigorous shaken by hand for 5 s. This digestion step was repeated 3 times with fresh enzyme solution. Cells were then passed through a 70 μm pore diameter cell strainer, pelleted at 300 rcf for 5 min and resuspended in DMEM 1885 with 10% fetal bovine serum. An equal volume of 0.05% trypsin–EDTA (15400054; Life technologies) was added and cells were incubated at 37 °C for 2 min. Before sorting, cells were pelleted, resuspended in DMEM 1885, 10% fetal bovine serum, and filtered again (35 μm).

### 2.9 RNA extraction and quantitative RT-PCR analysis

From *Gpr64*^*mCherry*^ reporter mice, RNA was extracted from 10,000 to 30,000 cells and DNase treated using a NucleoSpin RNA XS kit (Macherey–Nagel). RT-PCR was performed using SuperScript III Reverse Transcriptase (Invitrogen). Custom-designed StellARray qPCR arrays (Lonza Group) were assayed according to the manufacturer's instructions with SYBR Premix Ex Taq (TaKaRa) using a lightcycler480 (Roche). Relative expression was calculated using the delta–delta CT analysis method.

### 2.10 RNA-seq analysis

Five 24-week-old *Trpm5*^*GFP*^*:Gpr64*^*mCherry*^ reporter mice were euthanized and 14 cm of proximal small intestine was excised and treated as described above. RNA was extracted and DNase treated from 6,000 to 12,000 young tuft cells (*Trpm5*^*GFP*^-positive, *Gpr64*^*mCherry*^-negative), 56,000–70,000 mature tuft cells (*Trpm5*^*GFP*^-positive, *Gpr64*^*mCherry*^-positive) and 60,000 non-tuft background cells (*Trpm5*^*GFP*^-negative, *Gpr64*^*mCherry*^-negative) using a NucleoSpin RNA XS kit (Macherey–Nagel). Full length cDNA were generated from 2 ng of total RNA using the SMART-Seq v4 Ultra Low Input RNA kit for Sequencing (Clontech, Part number 634890) according to the manufacturer's instructions with 12 cycles of PCR for cDNA amplification by Seq-Amp polymerase (Clontech). We then used 600 ng of pre-amplified cDNA as input for Tn5 transposon tagmentation by the Nextera XT kit (Illumina, Part number FC-131-1096) followed by 12 cycles of library amplification. Following purification using Agencourt AMPure XP beads (Beckman Coulter, Part number A63882), the size, and concentration of library DNA were assessed on an Agilent 2100 Bioanalyzer. Libraries were then sequenced on an Illumina HiSeq4000 system as single-end 1 × 50 base reads. Image analysis and base calling were performed using RTA 2.7.3 and bcl2fastq 2.17.1.14. Reads were mapped onto the mm9 assembly of mouse genome using Tophat v2.0.14 [40] with bowtie v2.1.0 aligner. Gene expression was quantified using HTSeq v0.6.1 [41] and gene annotations from Ensembl release 67. Differential gene expression analysis was performed using R and DESeq2 v1.6.3 Bioconductor package [42], taking into account the pairing of samples from the same mouse. First, DESeqDataSetFromMatrix function was used to create a DESeqDataSet object from raw count data obtained using HTSeq, using the following design formula: ∼ Mouse + Cells, where Mouse corresponds to mouse number and Cells to the corresponding cell population. Then, differential expression analysis based on the Negative Binomial distribution was performed using DESeq function (estimation of size factors, estimation of dispersion, Negative Binomial GLM fitting and Wald statistics). Finally, results tables for the three comparisons between each pair of cell population were generated using results function, without Cooks cut-off nor independent filtering. Significantly differentially expressed genes were then selected using the following thresholds: p-value adjusted for multiple testing (using Benjamini and Hochberg method [43]) < 0.05 and |log2 Fold-Change| > 1.

### 2.11 Gene ontology enrichment analysis

Gene ontology (GO) enrichment analysis on the 22,189 identified genes of the young and mature tuft cells was carried out in R. As input, we used the log10-transformed RNA-seq DE p-values with inverted sign, multiplied by the sign of the log2-fold change, and used a two-sided Mann–Whitney–Wilcoxon test to compare the mean rank for genes in a GO geneset with the mean rank of all other genes. The test was carried out using GO genesets comprising at least 10 and at most 500 genes, resulting in 8222 tests. The resulting enrichment p-values were log10-transformed and their sign inverted for plotting. The statistical significance threshold, indicated by the horizontal dotted line in Figure 6C, was adjusted for testing across the 8222 genesets using the Bonferroni method, resulting in a cut-off of P < .05/8222.

### 2.12 Statistics

Data were visualized and tested for significance using Graph-Pad Prism 6 software. Error bars represent mean ± standard error of mean (SEM). Data were analyzed with non-parametric two-way ANOVA. Significance is defined as ∗: P < .05, ∗∗: P < .01, ∗∗∗: P < .001 and ∗∗∗∗: P < .0001 for all tests.

## 3 Results

### 3.1 Gpr64^*mCherry*^ expression in multiple organs including epididymis and parathyroid gland

**Figure 1:**
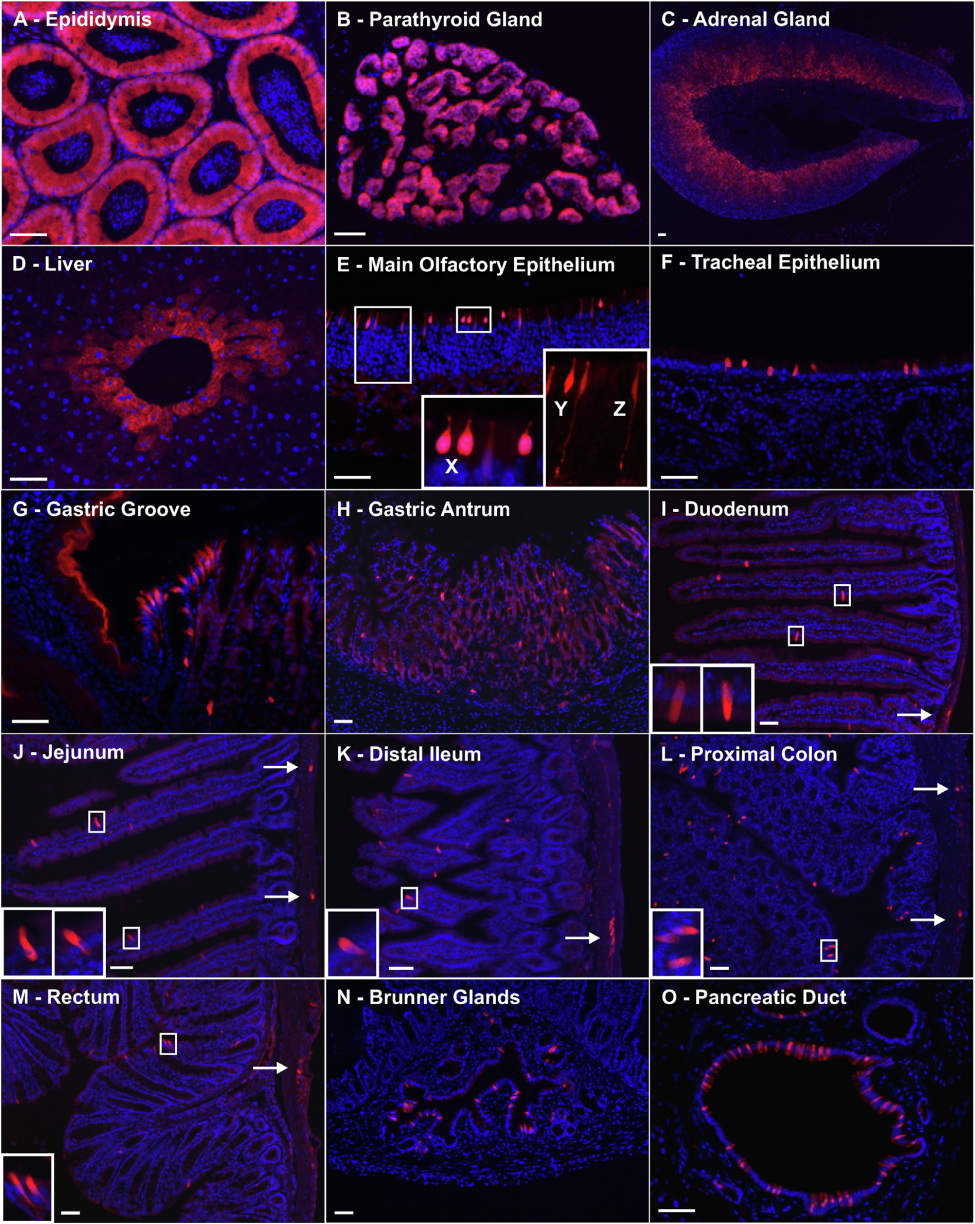
**Histological examination of the transgenic Gpr64-mCherry reporter mice** Representative fluorescence microscopy images showing *Gpr64*^*mCherry*^ fluorescence (Red) and DAPI nuclei staining (Blue) from A) epididymis, B) parathyroid gland, C) adrenal Gland, D) liver central vein, E) Main olfactory epithelium exhibited three morphologically distinct types of *Gpr64*^*mCherry*^-positive cells: Pear-shaped cells (X), flask-shaped cells (Y) and olfactory sensory neurons (Z), F) tracheal epithelium, G) pancreatic duct, H) gastric groove, I) gastric antrum, J) Brunner glands, K) duodenum, L) Jejunum, M) distal ileum, N, proximal colon and O) rectum. Arrows indicate mCherry fluorescence in myenteric plexi throughout the intestine. Insets show cells in higher magnification. Male mice n = 3. Bar = 50 μm.

To identify tissue sites of *Gpr64* expression, we used a transgenic mouse line, in which expression of the red fluorescent mCherry protein was under the control of the *Gpr64/Adgrg2* promoter (termed *Gpr64*^*mCherry*^). As expected, *Gpr64*^*mCherry*^ mice displayed intense fluorescence in epididymis and parathyroid, where Gpr64 previously has been described to be expressed [33,34] (Figure 1A,B). In the epididymal epithelium, the expression was strongest at the head and fading towards the tail (data not shown). Surprisingly, the cortex of the adrenal gland also showed mCherry expression with strong fluorescence in the zona reticularis and slightly lower fluorescence in the zona fasciculata fading towards the zona glomerulosa, which was devoid of red fluorescence (Figure 1C). Hepatocytes surrounding the central veins also displayed mCherry fluorescence (Figure 1D).

### 3.2 Gpr64^*mCherry*^ is highly expressed in chemosensory epithelial cells of the respiratory tract

In the respiratory tract, numerous, solitary, strongly red-fluorescent epithelial cells were observed in the *Gpr64*^*mCherry*^ mice (Figure 1E,F). In the olfactory epithelium three morphological distinct *Gpr64*^*mCherry*^-positive cell types were identified: pear-shaped cells, flask-shaped cells and typical olfactory sensory neurons (ONS) (Figure 1E, labeled X, Y, and Z, respectively) being most abundant in the main olfactory epithelium (MOE) of the posterior nasal cavity.

In the tracheal epithelium, solitary *Gpr64*^*mCherry*^-positive cells were observed whilst in the bronchioles further down the respiratory tract, no fluorescent cells were observed in the epithelium; however, in the subepithelia, *Gpr64*^*mCherry*^-positive cells with slender processes were observed (data not shown).

**Figure 2:**
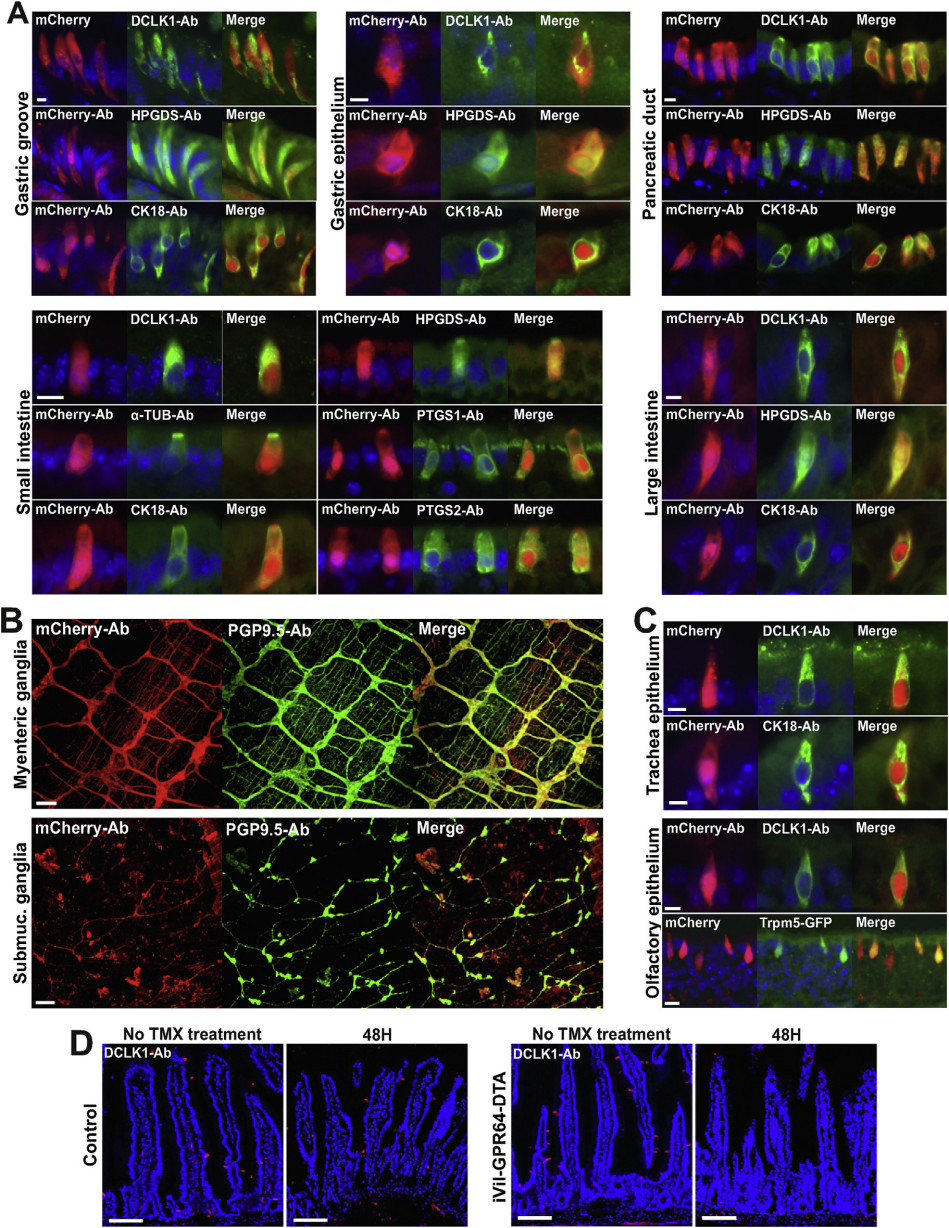
**Gastrointestinal Gpr64-mCherry positive cells are enteric nerves and tuft cells** Representative fluorescence microscopy images of *Gpr64*^*mCherry*^ tissue sections immunostained for selected markers specific for tuft cells, microvillous cells, and enteric nerves. A) GI tract *Gpr64*^*mCherry*^-positive cells (red) stained with doublecortin like kinase 1 (DCLK1), acetylated alpha Tubulin (AC-TUB), cytokeratin 18 (CK18), hematopoietic prostaglandin D synthase (HPGDS), prostaglandin D2 Synthase 1 (PTGS1) and prostaglandin D2 Synthase 2 (PTGS2) antibodies (green) and counterstained with DAPI (blue). Bar = 5 μm. B) *Gpr64*^*mCherry*^-positive submucosal and myenteric nerves immunostained with anti-PGP9.5 Bar = 50 μm. C) Respiratory tract *Gpr64*^*mCherry*^ cells stained with DCLK1 and CK18 antibodies (scale bar, 5 μm). Fourth panel displays olfactory epithelium isolated from the double transgenic *Trpm5*^*GFP*^*;Gpr64*^*mCherry*^ reporter mouse with both *Gpr64* promoter-driven mCherry and *Trpm5* promoter-driven GFP expression. Bar = 20 μm. Male mice n = 3. Ab, Antibody. D) Small intestinal sections from *GPR64*^*DTA*^ control mice (upper row) and *Vil-CreERT2;GPR64*^*DTA*^ mice (lower row) before and 48 h after tamoxifen induced cell ablation. Tuft cells visualized with anti-DCLK1 (red). Bar = 100 μm. Per group n = 6–8 mice.

The respiratory epithelium displayed very few cells immunoreactive for tuft cell marker proteins. In the tracheal epithelium, only a few cells were Dclk1 and to some extent CK18 positive. However, these cells also displayed *Gpr64*^*mCherry*^ fluorescence (Figure 2C). Likewise, in the olfactory epithelium, very few cells were positive for Dclk1 staining, but co-localized with mCherry expression (Figure 2C) suggesting that the majority of *Gpr64*^*mCherry*^-positive cells in the nasal cavity were not tuft cells. The *Gpr64*^*mCherry*^-positive olfactory sensing neurons were not further characterized here. The pear-shaped *Gpr64*^*mCherry*^-positive cells of the olfactory epithelium displayed morphological resemblance to the poorly characterized Trpm5-expressing, pheromone-sensing so-called microvillous cells [[44], [45], [46]]. Together, these results indicate that *Gpr64* is expressed in various chemosensory cells of the respiratory epithelium namely ONS, microvillous cells, and respiratory tuft cells.

### 3.3 Gpr64^mCherry^ expression in the GI tract

A cluster of brightly fluorescent *Gpr64*^*mCherry*^ -positive cells was observed at the gastric groove, i.e., the border between the forestomach and the corpus of the stomach, (Figure 1G). The outermost epithelial layer of the forestomach also displayed a surprisingly strong, uniform apparently non-cellular mCherry fluorescence, a trend that continued all the way up through the esophagus (data not shown). Importantly, distal to the gastric grove and throughout the rest of the stomach only relatively low or no background expression of mCherry was observed with few, scattered, brightly fluorescent cells (Figure 1H). Throughout the remaining intestinal tract, mCherry was observed both in solitary cells situated primarily in the epithelium of the villi as well as in clusters within the intestinal muscular wall suggesting expression in enteric ganglion cells (Figure 1I-M). This was confirmed by co-staining with the pan-neuronal marker PGP9.5 revealing co-localization with mCherry expression in both submucosal and myenteric ganglia (Figure 2B). The epithelium of the Brunner gland (Figure 1N) and pancreatic duct (Figure 1O) showed relatively high densities of scattered, brightly fluorescent, *Gpr64*^*mCherry*^-positive cells.

In conclusion, the histological examination of the *Gpr64*^*mCherry*^ reporter mice revealed mCherry fluorescence in scattered cells of the epithelium and in enteric neurons throughout the GI tract.

### 3.4 Gpr64^mCherry^ is highly expressed in tuft cells of the GI tract

Histological examination of the *Gpr64*^*mCherry*^ mice revealed brightly-fluorescent mCherry-positive cells located in respiratory and GI epithelia – with the characteristics of tuft cells. Immunohistochemical labeling for tuft marker proteins was applied and co-localization with mCherry expression was examined. Some antibodies required ‘antigen retrieval’ in order to bind their target. This procedure abolished the endogenous mCherry fluorescence, which was retrieved using specific mCherry-antibodies.

Immunohistochemical labeling revealed that the *Gpr64*^*mCherry*^ cells of the gastric groove, gastric epithelium, pancreatic duct, Brunner's glands, small and large intestine co-localized with the tuft cell markers such as Dclk1, Hpgds, and CK18 as shown in Figure 2A. Moreover in the small intestine, *Gpr64*^*mCherry*^-positive cells also co-localized with acetylated α-tubulin (Ac-Tub) and prostaglandin-endoperoxide synthase 1 and 2 (Ptgs1 and 2) (Figure 2A). Furthermore, we also tested for co-localization with enteroendocrine cell markers in the small intestine, but did not observe any co-localization of *Gpr64*^*mCherry*^ fluorescence and cholecystokinin, glucose-dependent insulinotropic peptide, secretin, Glucagon-like peptide-1 (GLP-1), peptide YY, neurotensin, somatostatin, serotonin nor substance P (data not shown).

To test if all small intestinal tuft cells expressed Gpr64, we used transgenic *Vil-CreERT2;GPR64*^*DTA*^ mice to conditionally ablate Gpr64-expressing epithelial cells upon tamoxifen-treatment. Tuft cells were visualized with anti-Dclk1. Two days after ablation, the small intestine was completely devoid of tuft cells (Figure 2D), which together with the immunohistological co-staining demonstrate that *Gpr64* is expressed in tuft cells of the GI tract.

### 3.5 GPCR profiling of mature Gpr64^mCherry^ intestinal tuft cells of the villi

**Figure 3:**
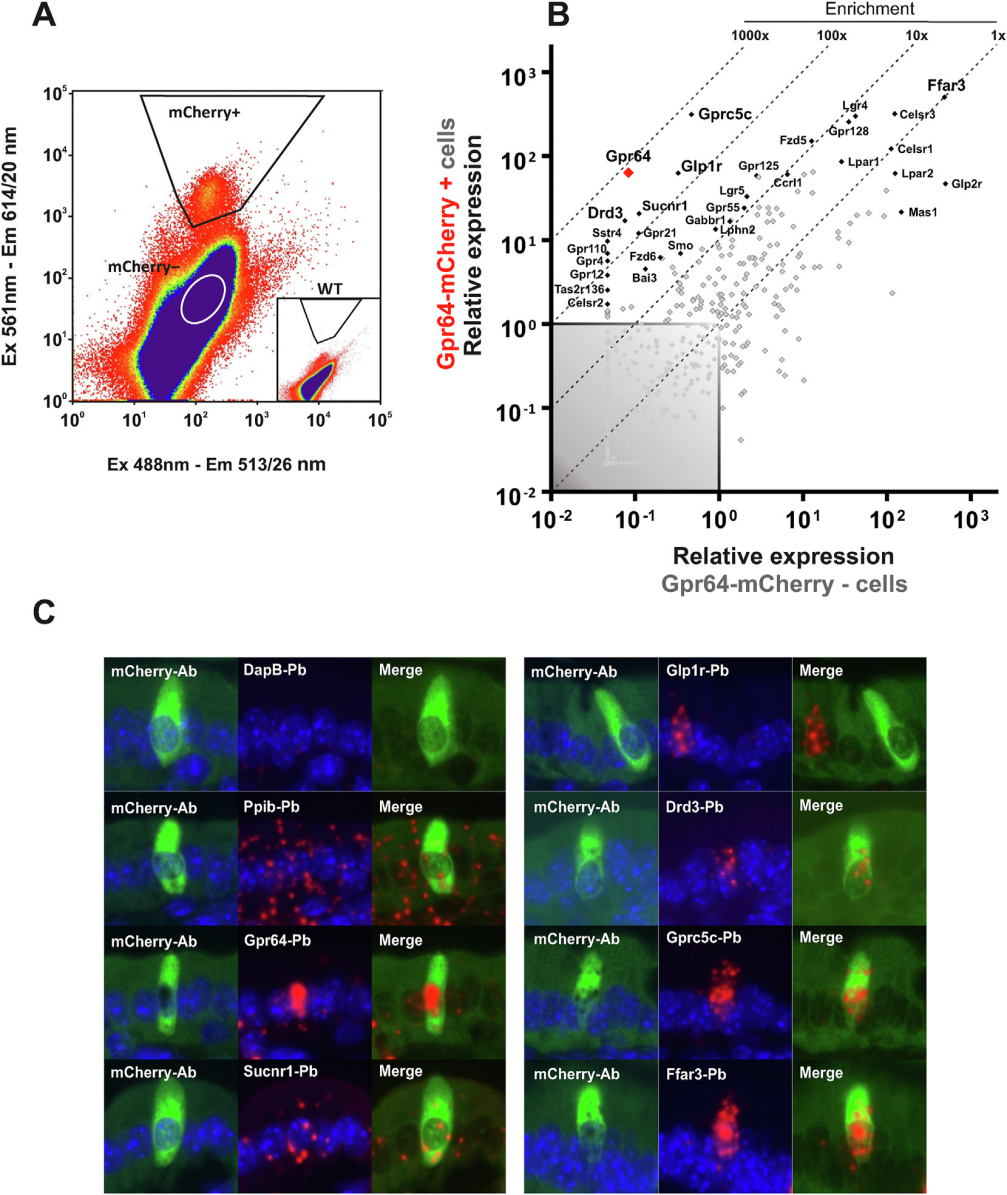
**Non-odorant GPCR expression profile of mature tuft cells** A) Representative FACS diagram showing gate (trapezoid) used for sorting *Gpr64*^*mCherry*^-positive tuft cells based on emission at 614 and 513 nm after excitation at 561 and 488 nm, respectively. B) Quantitative RT-PCR analysis of FACS-purified *Gpr64*^*mCherry*^-positive cells. The relative expression of 379 GPCRs in *Gpr64*^*mCherry*^-positive cells (y-axis) vs *Gpr64*^*mCherry*^-negative cells (x-axis). The enriched GPCRs are displayed with gene name. The remaining GPCR are shown as grey dots. N = 5, each consisting of cells derived from 3 pooled mice (total 15 male mice). C) Dual immunohistochemistry and *in situ* hybridization probing for selected GPCR's (Red fluorescence) on from *Gpr64*^*mCherry*^ duodenum sections. mCherry signal was retrieved using mCherry-specific antibodies (Green fluorescence). Each red dot represents a single stained mRNA transcript. Positive control probe: *Mus musculus*, Peptidylpropyl isomerase B (*Ppib*). Negative control probe: *Bacillus subtilis*, dihydrodipicolinate reductase (*DapB*). Ab: Antibody. Pb: *in situ* hybridization probe. Nuclei were visualized with DAPI counterstaining (blue). Male mice n = 3.

The GPCR repertoire of the intestinal tuft cell is poorly characterized. To investigate this, small intestinal *Gpr64*^*mCherry*^ mucosa was subjected to enzymatic digestion and the mCherry cells were purified by FACS (Figure 3A). The *Gpr64*^*mCherry*^-positive versus *Gpr64*^*mCherry*^-negative cells were analyzed for 379 non-odorant GPCRs by means of a qPCR array [47] (Figure 3B).

As expected, *Gpr64* was the most highly enriched receptor transcript, demonstrated by an almost 1000-fold enrichment in the *Gpr64*^*mCherry*^-positive cells compared to surrounding cells. A number of other GPCRs were also found to be selectively expressed in the *Gpr64*^*mCherry*^-positive cells including dopamine D3 receptor (*Drd3*), glucagon-like peptide 1 receptor (*Glp1r*), succinate receptor 1 (*Sucnr1*) and G-protein coupled receptor family C group 5 member C (*Gprc5c*). Interestingly, the short chain fatty acid receptor *Gpr41/Ffar3*, which previously has been identified in enteroendocrine cells and enterocytes [48] was also highly expressed in both *Gpr64*^*mCherry*^-positive and –negative cell populations.

To validate these results, a combined immunofluorescence and *in situ* hybridization technique was applied to *Gpr64*^*mCherry*^ duodenal sections. Focusing on the highly enriched receptors according to the qPCR analysis. After *in situ* hybridization, mCherry signal was retrieved using immunohistochemistry with antibodies raised against mCherry. As shown in Figure 3C, epithelial *Gpr64*^*mCherry*^-positive cells were found to contain transcript-staining for *Gpr64* as well as *Sucnr1* and *Drd3*. The *Gpr64*^*mCherry*^ cells did contain high numbers of stained *Gprc5c* and *Ffar3* transcripts, but unexpectedly, no mucosal *Gpr64*^*mCherry*^-positive cells contained any labeled *Glp1r* transcripts. We conclude that *Gpr64*^*mCherry*^-positive tuft cells of the small intestine express not only *Gpr64* as expected, but also *Sucnr1*, *Drd3*, *Gprc5c*, and *Ffar3*.

### 3.6 Gpr64 is expressed only in mature tuft cells of the villi as opposed to Trpm5

The *Gpr64*^*mCherry*^-positive tuft cells were primarily observed in the villus region, rather than being evenly distributed across the crypt–villus axis as would be expected for small intestinal tuft cells [49]. Previous studies have shown Trpm5 to be specifically expressed in basically all of small intestinal tuft cells throughout the crypt–villus axis [16,18]. To compare the *Gpr64*^*mCherry*^-positive tuft cells to a general tuft cell population and subsequently sort and characterize them, we crossed the *Gpr64*^*mCherry*^ mice with *Trpm5*^*GFP*^ mice yielding *Trpm5*^*GFP*^*:Gpr64*^*mCherry*^ double reporter mice and examined their small intestine.

**Figure 4:**
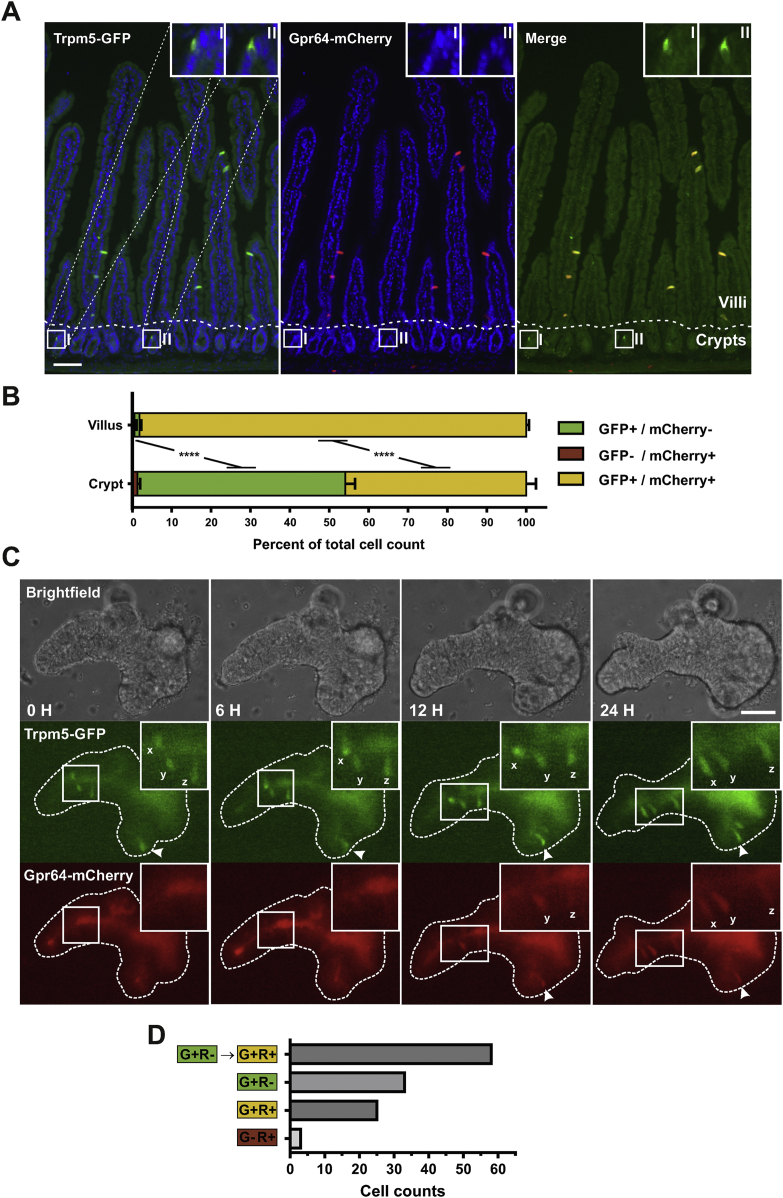
**Histological and organoid studies indicate sequential expression of Trpm5, and subsequently Gpr64, in maturing intestinal tuft cells** A) Representative fluorescence microscopy images of the double transgenic reporter *Trpm5*^*GFP*^*;Gpr64*^*mCherry*^ mouse duodenum showing *Trpm5* promoter-driven GFP fluorescence (Green), *Gpr64* promoter-driven mCherry fluorescence (Red) and DAPI nuclei staining (Blue). Merged picture on the right also contain magnification of *Trpm5*^*GFP*^-positive cryptal cells, but no visible *Gpr64*^*mCherry*^ fluorescence. Dashed line separates crypt (C) and villus (V) area. Bar = 50 μm. B) Quantification of *Trpm5*^*GFP*^ and *Gpr64*^*mCherry*^ co-localization in crypt and villus area. Normalized to total cell count of crypt or villus area. GFP signal was enhanced with antibodies. >300 counted cells per animal. Male mice n = 3. Data tested with 2-way ANOVA. C) Representative timelapse images of intestinal *Trpm5*^*GFP*^*;Gpr64*^*mCherry*^ organoid treated with recombinant Il-4 and Il-13. Brightfield, *Trpm5*^*GFP*^ fluorescence and *Gpr64*^*mCherry*^ fluorescence images were captured at timepoint: 0, 3, 6, 15 h. Initially, three *Trpm5*^*GFP*^-fluorescent tuft cells are observed (tagged: x,y and z) with no evident mCherry fluorescence. After 6–24 h, a gradual increase in *Gpr64*^*mCherry*^ fluorescence is observed in the three *Trpm5*^*GFP*^-positive tuft cells. Arrowhead indicate another cell first expressing GFP and then subsequently express mCherry. Insets show cells in higher magnification. Bar = 50 μm. D) Quantification of sequential expression of GFP and mCherry fluorescence in *Trpm5*^*GFP;*^*Gpr64*^*mCherry*^ organoids. Forty-five organoids containing 119 fluorescent cells were monitored for up to 60 h. Of the 119 cells, 58 cells were initially *Trpm5*^*GFP*^-positive/*Gpr64*^*mCherry*^-negative and then became *Trpm5*^*GFP*^-positive/*Gpr64*^*mCherry*^-positive. Average time from first detected GFP fluorescence to first detected mCherry fluorescence: 10.8 h ± 1.7 SEM. G + R- = Cell displaying *Trpm5*^*GFP*^-positive and *Gpr64*^*mCherry*^-negative fluorescence. G + R+ = Cell displaying *Trpm5*^*GFP*^-positive and *Gpr64*^*mCherry*^-positive fluorescence. G-R+ = Cell displaying *Trpm5*^*GFP*^-negative and *Gpr64*^*mCherry*^-positive fluorescence.

In the villus region of the duodenum, all of the *Trpm5*^*GFP*^-positive cells co-localize with *Gpr64*^*mCherry*^ fluorescence (Figure 4A). However, in the crypt region, we detected *Trpm5*^*GFP*^-positive cells without any apparent *Gpr64*^*mCherry*^ fluorescence. The degree of overlap between *Trpm5*^*GFP*^ and *Gpr64*^*mCherry*^ was quantified in the villus and crypt region as shown in Figure 4B. In the villus region, 98% of the detected cells display both *Gpr64*^*mCherry*^ and *Trpm5*^*GFP*^ fluorescence; however, in the cryptal area these double-expressing cells only account for 46% of the *Trpm5*^*GFP*^-positive cells. Thus, while half of the cryptal tuft cells express only *Trpm5*^*GFP*^, almost all villus tuft cells express both *Gpr64*^*mCherry*^ and *Trpm5*^*GFP*^, which indicates that Gpr64 is a marker of mature tuft cells.

To study the likely upregulation of *Gpr64*^*mCherry*^ in maturing tuft cells more dynamically, we generated duodenal organoids from *Trpm5*^*GFP*^*:Gpr64*^*mCherry*^ mice, which allowed us to track the development of the green and red fluorescence in cells over time. To promote tuft cell hyperplasia, Il-4 and Il-13 was administered to the organoids. The organoids displayed usual morphology as described for small intestine organoids, with well-defined crypt and villus domains [50]. Figure 4C shows an example of an organoid time-lapse experiment. Here, *Trpm5*^*GFP*^-fluorescent tuft cells were visible at the beginning of the experiment with no evident *Gpr64*^*mCherry*^ fluorescence. Over the course of 6–24 h, the appearance and gradual increase in *Gpr64*^*mCherry*^ fluorescence was observed in the initially only *Trpm5*^*GFP*^-positive tuft cells. To quantify this phenomenon, 119 fluorescent cells distributed across 45 organoids were monitored for up to 60 h. As seen in Figure 4D, of the 119 monitored tuft cells, we observed 58 cells initially showing only GFP fluorescence and subsequently displayed mCherry fluorescence. From the start until the end of the time-lapse experiment, 33 cells exhibited only *Trpm5*^*GFP*^ expression, while 25 cells displayed both *Trpm5*^*GFP*^ and *Gpr64*^*mCherry*^ fluorescence. The average time span from the detection of *Trpm5*^*GFP*^ signal to the first *Gpr64*^*mCherry*^ detection was 10.8 h (Figure 4D).

Having generated the double Gpr64/Trpm5 reporter mice, we reexamined the nasal, flask-shaped, *Gpr64*^*mCherry*^-positive cells and observed a complete overlap between GFP and mCherry fluorescence (Figure 2C). This confirms that *Gpr64* is expressed in microvillous cells of the nasal epithelium, which previously have been shown to express *Trpm5* [44].

Concerning the GI tract, it is concluded that in contrast to the general tuft cell marker Trpm5, Gpr64 is a marker for mature tuft cells located in the intestinal villi which means that the Gpr64^mCherry^ reporter can be used to identify and characterize mature versus young tuft cells.

### 3.7 RNA-seq analysis on young and mature intestinal tuft cells

**Figure 5:**
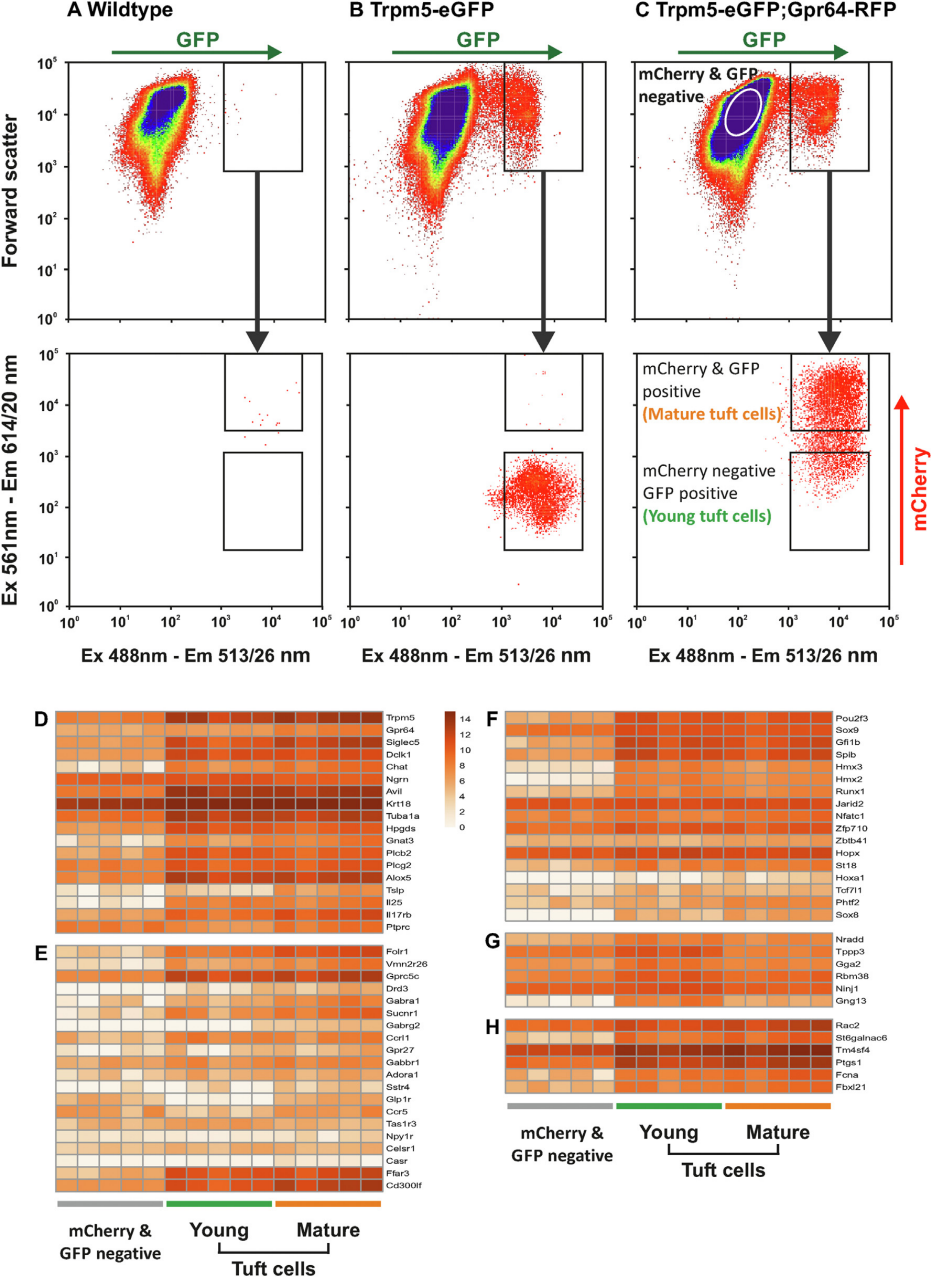
**RNA-seq reveal tuft cell signature genes and a dynamic chemosensory GPCR and TF expression in maturing intestinal tuft cell** FACS-gating of proximal small intestine preparations from A) WT, B) *Trpm5*^*GFP*^ reporter, C) *Trpm5*^*GFP*^*;Gpr64*^*mCherry*^ reporter mice. Young tuft cells (*Trpm5*^*GFP*^-positive, *Gpr64*^*mCherry*^-negative), mature tuft cells (*Trpm5*^*GFP*^-positive, *Gpr64*^*mCherry*^-positive) and non-tuft background cells (*Trpm5*^*GFP*^-negative, *Gpr64*^*mCherry*^-negative) were FACS-purified from the proximal small intestine of transgenic *Trpm5*^*GFP*^*;Gpr64*^*mCherry*^ reporter mice for mRNA sequencing. Heatmap displays the log2 (x+1) normalized expression of D) canonical tuft cell marker genes, E) selected non-odorant GPCR, F) selected TFs, G) tuft-1 markers, H) tuft-2 markers genes in the young-, mature tuft cells and background cell populations. Mice n = 5.

To identify unique and shared features of the young (*Trpm5*^*GFP*^-positive/*Gpr64*^*mCherry*^-negative) and mature (*Trpm5*^*GFP*^-positive/*Gpr64*^*mCherry*^-positive) intestinal tuft cells, we enzymatically digested *Trpm5*^*GFP*^*:Gpr64*^*mCherry*^ proximal small intestine and FACS-purified (Figure 5C) young (*Trpm5*^*GFP*^-positive/*Gpr64*^*mCherry*^-negative), mature (*Trpm5*^*GFP*^-positive/*Gpr64*^*mCherry*^-positive) tuft cells and surrounding cells (*Trpm5*^*GFP*^-negative/*Gpr64*^*mCherry*^-negative) for subsequent mRNA sequencing. As a control of FACS-gating, we compared with WT (Figure 5A) and *Trpm5*^*GFP*^ (Figure 5B) mice.

Transcriptomic analysis of young (*Trpm5*^*GFP*^-positive/*Gpr64*^*mCherry*^-negative) and mature (*Trpm5*^*GFP*^-positive/*Gpr64*^*mCherry*^-positive) tuft cells revealed a strong enrichment for canonical tuft cell signature genes compared to other intestinal cells (Figure 5D). Furthermore, *Trpm5* was enriched in both young and mature tuft cells, while *Gpr64* was primarily expressed in the mature tuft cells.

As seen in Figure 5E, RNA-seq analysis showed increased expression of the GPCRs; *Drd3*, *Glp-1r*, *Sucnr1*, *Gprc5c*, *Ffar3,* and *Sstr4* in the mature tuft cells supporting previous findings by qPCR. RNA-seq analysis revealed additional receptors that were expressed in young and mature tuft cells and that the degree of expression changed between the two groups. While *Gprc5c*, *Ccrl1/Ackr4*, *Gabbr1*, *Adora1*, *Celsr1*, and *Ffar3* expression remained largely unchanged between young and mature tuft cells, *Vmn2r26*, *Drd3*, *Gabra1*, *Sucnr1*, *Gabrg2*, *Gpr27*, *Sstr4*, *Glp-1r*, *Ccr5*, *Npy1r,* and *CasR* expression was markedly higher in mature tuft cells compared to young with the exception of *Tas1r3* being higher in young tuft cells. Notably, the non-GPCR *Cd300lf* [51] was enriched in both young and mature tuft cells, while *Folr1* expression was higher in mature tuft cells compared to young.

Likewise, RNA-seq analysis also showed transcription factors (TFs) expression in young and mature tuft cells (Figure 5F). *Pou2f3* [13,20,52] and *Sox9* [13,53]; *Gfi1b* [13,18,19,52]; *Spib*, *Hmx3*, *Hmx2*, *Runx1*, *Jarid2*, *Nfatc1*, *Zfp710* and *Zbtb41* [52]; and *Hopx* [53] have previously been recognized as TFs for murine intestinal tuft cells and were also observed in our young and mature tuft cells. However, both young and mature tuft cells expressed *Klf5*, *Hopx*, *Jarid2*, and *Zbtb41* to the same degree as the non-tuft cell population (*Trpm5*^*GFP*^-negative/*Gpr64*^*mCherry*^-negative). Interestingly, we also identified expression of TFs: *St18*, *Hoxa1*, *Tcf7l1*, *Phtf2,* and *Sox8* in the young and mature tuft cells, which have not previously been reported in tuft cells.

Recently, Haber et al. described two subsets of tuft cells; Tuft-1 and Tuft-2 cells, based on different transcriptional profiles [52]. In our analysis, signature genes of Tuft-1 were observed to be generally more enriched in young tuft cells, compared to mature tuft cells and non-tuft cells (Figure 5G). However, signature genes for Tuft-2 were enriched in both young and mature tuft cells (Figure 5H) compared to non-tuft cells.

**Figure 6:**
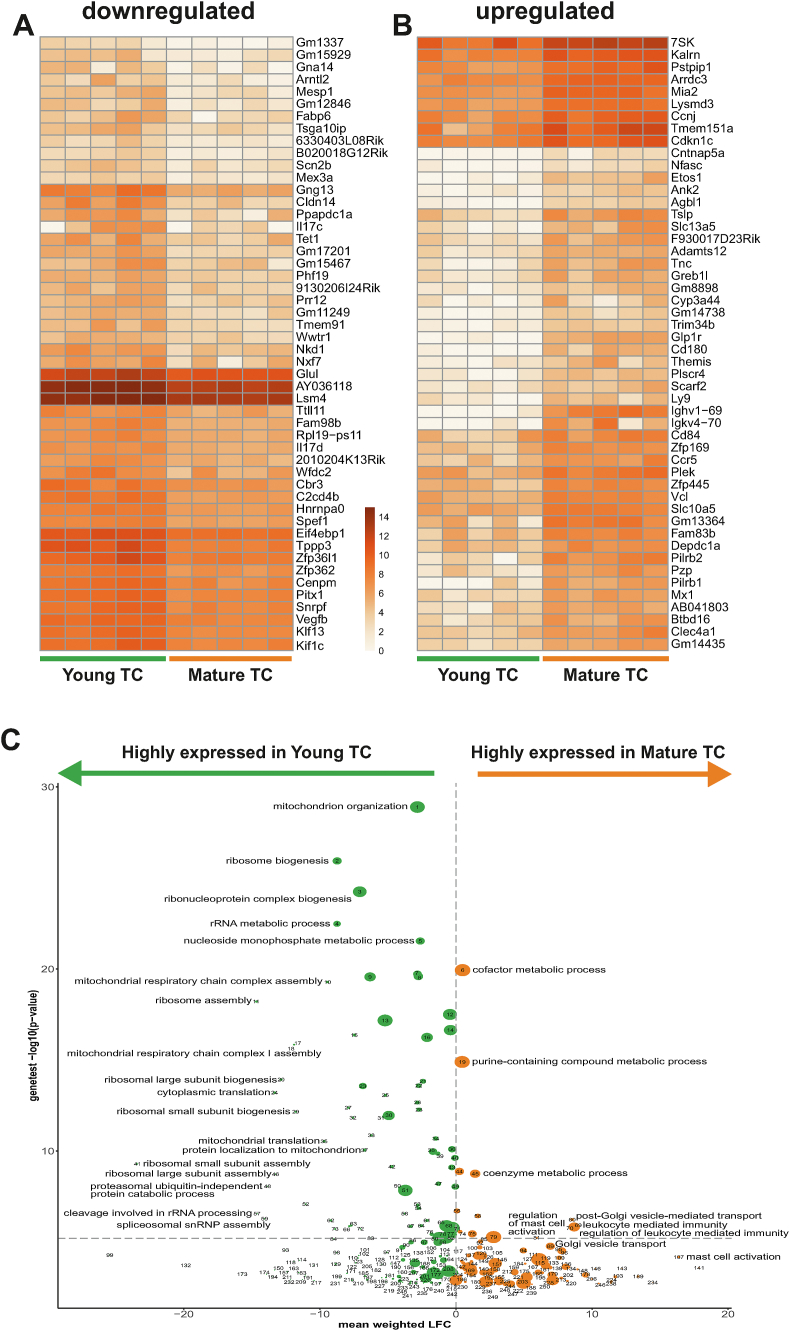
**Comparative transcriptional analysis of young vs. mature intestinal tuft cells** Heatmap depicting the log2 (x+1) normalized expression of 50 genes with the highest log2 fold A) downregulation or B) upregulation between young tuft cells (*Trpm5*^*GFP*^-positive, *Gpr64*^*mCherry*^-negative) and mature tuft cells (*Trpm5*^*GFP*^-positive, *Gpr64*^*mCherry*^-positive) from *Trpm5*^*GFP*^*;Gpr64m*^*Cherry*^ proximal small intestine (all plotted genes had statistically significant log2 fold changes in mature versus young cells, P < 0.01). C) Volcano plot. The vertical dimension represents the log10-transformed p-values (with inverted sign) from a two-sided Mann–Whitney–Wilcoxon geneset enrichment test (Materials and Methods). The horizontal dimension represents the weighted mean of the log2 fold change in expression of the geneset genes in old versus young tuft cells, where each gene's contribution is weighted by the log-transformed p-value (with the sign reversed) corresponding to the log2 fold change. Thus green bubbles left of zero represent genesets which are downregulated in old versus young tuft cells, and red bubbles represent upregulated genesets. The size of the bubble represents the number of genes in the geneset. Mice n = 5.

To explore the genes that exhibited the highest degree of regulation between young and mature tuft cells, we examined the 50 genes with the highest log2 fold down- (Figure 6A) and upregulation (Figure 6B) between the two groups, for all of which the log2 fold change was statistically significant (P < 0.01).

Cytokines *Il-17c* and *Il-17d* and TFs *Arntl2*, *Mesp1*, *Phf19*, *Pitx* and *Klf13* were increased in young tuft cells compared to mature (Figure 6A).

To understand the biological features, we performed a GO enrichment analysis using the DE results from 22,189 identified genes of the young and mature tuft cells and plotted the enriched genesets as depicted in Figure 6C, with all nominally significant GO terms (not adjusting for multiple testing) listed in Table S3. A surprisingly clear difference between mature *Gpr64*^*mCherry*^-positive tuft cells and young *Gpr64*^*mCherry*^-negative tuft cells was observed in the GO-terms associated with a positive or negative log2 fold change between them (Figure 6C). Thus, whereas many GO genesets were identified as overlapping with genes that were upregulated in the young tuft cells (annotated in green to the left in the volcano plot) only very few GO-terms were significantly overlapping with genes upregulated in the mature tuft cells (red, on the right). Thus, young tuft cell displayed a marked enrichment for multiple GO terms related to, for example biosynthesis and assembly of constituent parts of ribosome subunits, mitochondrion organization, and rRNA processing. Mature tuft cells displayed only a modest enrichment for few GO terms primarily related to ‘mast cell activation’ and ‘Golgi vesicle transport’. These clear differences in gene expression patterns (Figure 6C) together with the histological identification demonstrate the power of *Gpr64*^*mCherry*^ to differentiate between mature, villus-located versus young, crypt-located tuft cells (Figure 4).

## 4 Discussion

By use of a novel *Gpr64*^*mCherry*^ mouse strain, we herein identified the adhesion receptor GPR64 as a novel component of specific chemosensory and glandular cells throughout the body beyond the limited known expression in the epithelium of the epididymis and in the parathyroid gland [33,34]. Thus, GPR64 is expressed in hepatocytes surrounding the central veins and in the zona reticularis of the adrenal gland, as well as in both sensory neurons and microvillus cells of the olfactory epithelium, and in enteric nerves. Surprisingly, in the respiratory epithelium and throughout the GI tract GPR64 was expressed specifically in the elusive sensory, immuno-regulatory tuft cells. Most significantly, in the intestine we found GPR64 to be expressed selectively in mature tuft cells of the villi as opposed to young tuft cells of the crypts. This differential expression enabled us to identify the different gene expression repertoire of mature vs. immature tuft cells including novel transcription factors and receptors. Importantly, the selective expression of GPR64 in mature tuft cells suggests an important role for this ADGR in tuft cell function.

### 4.1 Reliability of the Gpr64^mCherry^ reporter

As no reliable antibodies for murine GPR64 are available (unpublished), we were not able to confirm the presence of GPR64 protein at sites of the *Gpr64*^*mCherry*^ reporter expression. However, we could confirm high expression and enrichment of *Gpr6*4 mRNA in FACS-purified intestinal *Gpr64*^*mCherry*^ fluorescent cells by means of qPCR and RNA-seq analysis. Furthermore, *in situ* hybridization also demonstrated *Gpr6*4 mRNA expression in intestinal mucosal *Gpr64*^*mCherry*^ positive cells using antibodies against mCherry. These data, together with the expression of *Gpr64*^*mCherry*^ in epididymal epithelium and in the parathyroid, underlines the reliability of the *Gpr64*^*mCherry*^ reporter for GPR64 receptor expression.

### 4.2 The role of GPR64 in chemosensory and secretory cells

Like many other ADGRs, GPR64 is still an orphan receptor with unknown ligand. It is however likely that in analogy with other ADGRs, GPR64 recognizes some extracellular macromolecule through its large N-terminal fragment. In general, ADGRs are activated through the removal of the already auto-cleaved but still associated N-terminal domain from the 7TM domain, after which the now exposed small N-terminal, so-called ‘stachel peptide’ extension on TM-I acts as a tethered agonist for activation of the 7TM domain [54]. Originally, we published that GPR64 in contrast to most ADGRs signals with high constitutive activity through activation of SRE and NFκB transcriptional activation conceivably through G12/13 and Gq/11 [31]. However, it was recently reported that removal of the N-terminal segment from GPR64 results in increased Gs signaling including downstream CREB activation from the remaining 7TM domain and interaction of this domain with the CasR in the parathyroid [34]. Thus, it is possible that binding of the large N-terminal segment of GPR64 to a yet unidentified macromolecule changes its signaling pathways from constitutive G12/13 and Gq to Gs; however, this remains to be confirmed. Interestingly, like GPR64 CasR was upregulated in mature tuft cells compared to young and could therefore potentially co-function with GPR64 also in these cells.

Knock-out studies suggest that GPR64 monitors the luminal content of the epididymis and modulates fluid uptake in the efferent tubules [35]. This would indicate that GPR64 could function in a similar fashion in other organ cavities, i.e. as an extracellular sensor monitoring and maintaining fluid homeostasis. Another possibility is that GPR64 could play a role in cell polarization as the majority of the *Gpr64*^*mCherry*^-positive cells identified in the present study throughout the body are highly polarized; and, other ADGR's have been implicated in coordinated spatial arrangements of organs, tissues, and cells. This includes the structurally similar GPR56 (ADGRG1), which is critical for cerebellar morphogenesis. GPR56 interacts with collagen III and suppresses neuronal migration during cerebral cortex development [55] giving Gpr56 KO mice cerebral cortex malfunction due to migration of neurons beyond the pial basement membrane [56]. Similarly, CELSR (ADGRC1) which is upregulated in both young and mature tuft cells is a key protein involved in coordination of planar cell polarity [57,58]. The fluid buildup seen in previous Gpr64 knock-out studies, could therefore possibly be due to lack of proper polarization of the fluid absorbing cells in the epididymal efferent duct.

### 4.3 Dynamic expression pattern of maturing intestinal tuft cells

Trpm5 is well established as a general marker for intestinal tuft cells [16,18]. Conversely, mCherry under the control of the *Gpr64* promoter was only expressed in tuft cells of the intestinal villus and not in the crypts. Thus, the *Trpm5*^*GFP*^*:Gpr64*^*mCherry*^ double reporter enabled us to isolate mature versus young tuft cells and thereby identify changes in the transcriptional repertoire of tuft cells during their differentiation and migration from crypt to villus. A testament to that is the apparent upregulation of many GPCRs - besides GPR64 - and change in expression of transcription factors between young and mature tuft cells. In this way we were able to identify transcription factors, which to the best of our knowledge not previously have been associated with tuft cell differentiation, i.e. St18, Hoxa1, Tcf7l1, Phtf2, and Sox8 being upregulated and Arntl2, Mesp1, Phf19, Pitx, and Klf13 being downregulated in mature versus young tuft cells.

In their recent, pioneering single cell transcriptomic study of intestinal epithelial cells, Haber and coworkers distinguished between two subsets of intestinal tuft cells, tuft-1 and tuft-2 cells, based on PCA analysis of their transcriptional signature genes [52]. Interestingly, many genes enriched in tuft-1 cells were also observed to be enriched in our young tuft cells compared to the non-tuft cell and mature tuft cell population. However, signature genes for tuft-2 cells were enriched in both young and mature tuft cells. Like our young and mature tuft cells represent a temporal, differential segregation in the continually developing intestinal tuft cell population, we speculate that tuft-1 and tuft-2 cells may represent the same, albeit later in the differentiation process. Thus, tuft-1 marker genes could possibly be characteristics of an even younger tuft cell population than the one presented in this study and therefore primarily match our young tuft cells, while the tuft-2 marker genes would be found in both our young and mature tuft cells. Further studies are required to verify whether this is true.

Our young tuft cells were rich in GO term genes related to mitochondrial and ribosomal formation and organization and mRNA transcription, processing and splicing in accordance with a general, maturing cell type in the process of developing its bio-machinery. Conversely, the mature tuft cells were surprisingly characterized by a more modest number of GO terms encompassing for example Golgi vesicular transport and mast cell activation’. The fact that multiple genes and gene families are downregulated and that we do not observe a concomitant upregulation of a large number of other genes and gene families – i.e. that the volcano plot is skewed to the left - could make sense in a scenario where the mature, fully differentiated tuft cells probably are rather quiescent until challenged by intestinal pathogens only maintaining a basic output of cytokines, including Il-25, and an active vesicular transport.

### 4.4 Intestinal tuft cell receptors and pathogen interplay

Recent studies have established that mouse intestinal tuft cells initiate and drive the host defense response against parasitic- and protozoa infections [59]. However, how tuft cells detect harmful gut microbiota is unclear.

Recently, the microbial metabolite succinate and its receptor SUCNR1/GPR91 have been demonstrated by several groups to be important for tuft cell detection of luminal pathogens [20,21,60]. Although succinate is generated under normal physiological conditions, succinate concentrations are relatively low because it normally is efficiently converted to propionate by the gut microbiota. However, succinate concentrations can rise considerably in the intestinal lumen upon changes in gut motility, antibiotic treatment, and parasitic infection [61,62]. The succinate sensor GPR91 is highly expressed on intestinal tuft cells [16] and has been demonstrated to be vital in the initiation of the tuft cell-ILC2 driven immune response to, for example tritrichomonad infections [20,21,60]. Interestingly, GPR91 is also expressed on other cell types associated with type-2 immunity such as dendritic cells [63] and M2 macrophages [64].

In the present study, we confirm the enrichment of Sucnr1 in intestinal tuft cells and identify several other metabolite GPCRs, which could be potentially involved in monitoring of microbial activities in the gut lumen and overall intestinal health. One of these is the SCFA receptor FFAR3/GPR41 which also previously has been reported to be expressed in tuft cells, however with an unclear role [20]. SCFAs generated from bacterial fermentation of complex carbohydrates constitute an important energy source in particular for the intestinal metabolism itself [65], but SCFAs also function as important signaling metabolites being sensed by specific GPCR sensors expressed on enteroendocrine cells, enteric neurons and enteric leukocytes [48] but also affecting electrolyte secretion, smooth muscle contraction and cell growth [[66], [67], [68]]. Furthermore, receptors for SCFA have been shown to be essential for the mediation of intestinal inflammation [69]. Similar to the expression of Sucnr1, the expression of Ffar3/Gpr41 would enable the intestinal tuft cells to monitor the metabolic activity of the gut microbiota and potentially modulate the immune system accordingly.

We also identified the folate receptor (Folr1) in the intestinal tuft cells. Many bacterial species residing in the human GI tract are capable of synthesizing folate and elevated serum concentrations of folate have been linked to bacterial overgrowth in the upper small intestine [70,71]. This suggests that small intestinal tuft cells may be capable of detecting such pathogenic microbial expansions.

An interesting novel finding was that the D3 dopamine receptor is enriched in the intestinal tuft cells. In fact, substantial levels of dopamine are found in the lumen of the healthy gut [72,73] although its origin is somewhat unclear. Dopamine is produced by intrinsic enteric dopaminergic neurons [74,75] but also by non-neuronal, epithelial cells [76,77]. These epithelial cells apparently take up the dopamine precursor L-3,4-dihydroxyphenylalanine (l-DOPA) from the gut lumen and via aromatic amino acid decarboxylase (AADC) [76] convert it to dopamine, which diffuse out of the cells [77]. Interestingly, in patients with Crohn's disease and ulcerative colitis, as well as in TNBS colitis models, dopamine levels are markedly reduced, whereas the levels of the dopamine precursor, l-DOPA, are elevated suggesting a reduced activity of AADC in the epithelial cells or loss of nerves fibers caused by the mucosal inflammation [78,79]. The levels of GI dopamine therefore seemingly relate to overall health of the mucosa in the GI tract and could be sensed by D3 receptors expressed on the tuft cells.

In accordance with Wilen et al., we report that intestinal tuft cells - both young and mature - express Cd300lf, a murine norovirus (MNoV) receptor. Thus, the intestinal tuft cells appear to be the epithelial target cell of MNoV infection which is substantiated by IL-4 and IL-13 induced tuft cell hyperplasia, which amplify norovirus infection. Ironically, MNoV thus appears to exploit the intestinal tuft cells as means of immune evasion to promote viral infection during tuft cell hyperplasia [51].

We found Tas1r3 to be slightly enriched in young tuft cells, compared to other epithelial cells and mature tuft cells. Howitt et al. recently showed intestinal tuft cells to express Tas1r3, particularly in the ileum, and that Tas1r3-deficiency resulted in a reduced number of tuft cells in steady state, but also severely impaired antiparasite immunity when challenged with the protozoa *Tritrichomonas muris* or succinate [80]*.* The functional implications of this apparent upregulation in young tuft cells remains to be determined; however, it could suggest that the young tuft cells could act as a functionally distinct tuft cells subpopulation modulating differentiation or maintenance of the overall intestinal tuft cell population.

Most interestingly, GPR64 may itself as a receptor play a role in the tuft cells. In this connection it should be noted that GPR64 in polarized cells is expressed at the apical membrane [33], Thus, GPR64 in the mature tuft cells could potentially, through its large, highly O-glycosylated N-terminal domain, be involved in recognition and binding of luminal pathogens. A common feature of pathogenic microbes is their attachment to host glycosaminoglycans (GAG). Thus, it could be speculated that removal of the N-terminal domain of GPR64 as a result of binding of a pathogenic microorganism would activate or change the signaling of the receptor and thereby be involved in activation of mature tuft cells and thereby the type-2 immune reaction. This notion, however, remains to be proven.

### 4.5 Intestinal tuft cell crosstalk with immune system and ENS

Similar to the newly described ILC2-tuft cell circuit, our transcriptional analysis of maturing intestinal tuft cells indicates additional chemokine crosstalk with the intestinal immune system. Both young and mature intestinal tuft cells appear to express Ccrl1/Ackr4, which is an atypical receptor for CCL19, CCL21, and CCL25 important for leukocyte migration [81]. Interestingly, the young intestinal tuft cells had an upregulated expression of cytokines Il-17c and Il-17 d, which we are the first to report. Il-17c has been shown to serve a critical role in maintaining mucosal barrier integrity [82] and mediating mucosal immunity to intestinal pathogens [83] and IL-17 d has been observed to be important in virus surveillance [84]. This observation hints to a dynamic communication with the intestinal immune cells and the maturing tuft cells.

The enteric nervous system may also help to modulate intestinal tuft cells function. In agreement with previous observation, we found that intestinal tuft cells express the γ-aminobutyric acid (GABA) receptors: Gabra1, Gabrg2, and Gabbr1; however, we additionally observed enrichment of Drd3 and Npy1r in mature intestinal tuft cells.

## 5 Conclusion

The identification of GPR64 as a selective marker of mature intestinal tuft cells has potential functional implications related to the fact that it is an ADGRs, which generally are known to be activated though recognition of large biomolecules, which in the case of tuft cells could be speculated to possibly be pathogens. Importantly, the *Gpr64*^*mCherry*^ in combination with the *Trpm5*^*GFP*^ reporter provides a unique tool to further study tuft cell differentiation and activation in its role in type 2 immunity.

## Author contributions

K.V.G. and T.W.S. conceptualized the study and K.V.G. drafted the manuscript and T.W.S., K.L.E., S.T., J.J.T., M.S.E. C.K., G.G., S.O. edited the manuscript. S.T. designed and generated the transgenic reporter mice and performed the initial analysis of these. K.V.G. and S.T. designed, performed and analyzed the histology and immunofluorescence studies. K.V.G. designed, performed and analyzed the *in situ* hybridization studies. S.T. designed, performed and analyzed the *in vivo* diphtheria-toxin cell ablation studies. K.V.G. and N.P. designed, performed and analyzed intestinal organoid studies. K.V.G., K.L.E. and M.S.E. designed and performed the FACS-purification and qPCR analysis and interpreted results. C.K., C.V. and G.G. performed RNA-sequencing analysis and J.J.T., K.L.E., and K.V.G interpreted the results. K.V.G., K.L.E., T.W.S., M.S.E., S.T., G.G., and S.O. interpreted the results as a whole, provided important contributions to the manuscript and defined the main conclusions. All authors approved the final version of the manuscript.

## Funding

The project was supported by Challenge Grant NNF140C0013655 from the Novo Nordisk Foundation (T.W.S., S.O. and G.G.). The Novo Nordisk Foundation Center for Basic Metabolic Research (www.metabol.ku.dk) is supported by an unconditional grant, NNF10CC1016515 from the Novo Nordisk Foundation to University of Copenhagen. K.V.G. was supported by the Carlsberg Foundation. Sequencing was performed by the IGBMC Microarray and Sequencing platform, a member of the ‘France Génomique’ consortium (ANR-10-INBS-0009).

## Appendix A Supplementary data

Multimedia component 1 Figure S1. **Schematic overview of the generation of the BAC-based *Gpr64***^***mCherry***^**and GPR64**^**DTA**^**transgene** A bacterial artificial chromosome (BAC RP23-232E24) carrying the complete GPR64 gene was modified by Red/ET-recombination to insert either a A-B) cassette carrying the mCherry cDNA followed by a polyadenylation signal and an FRT-flanked ampicillin resistance cassette (β-lactamase) to generate the GPR64-mCherry transgene or C-D) a cassette consisting of a floxed eGFP and DTA construct into exon 3 of the GPR64 coding sequence to generate the GPR64-eGFP-DTA transgene. C) The construct contains a splice acceptor donor site, the loxp sequence and the eGFP with a splice acceptor site. The expression of the eGFP is stopped by a polyadenylation site (pA). Directly downstream of the eGFP is a Neomycin expressing cassette (Neo) driven by a phosphoglucokinase (PGK) promoter end stopped by a triple SV40 plolyadenylation site (3xSV40pA). The eGFP /Neo construct is flanked by the two loxp-sites for Cre recombination. Downstream of the second loxp site is the diphtheria toxin A cassette (DTA) located and terminated ba a bovine growth hormone polyadenylation site (polyA). For selection of the recombined BAC in bacteria a FRT-flanked ampicillin resistance cassette (Amp) is located downstream of the DTAn casette. This construct is flanked by a 5 prime and 3 prime homology arm (HA) to allow Red/ET recombination. The partial endogenous locus of the GPR64 gene ranging from exon 3 to 9 is shown below. The regions of recombination are indicated. D) Detailed sequence showing the recombination sites for the 3′ and 5′ homology arms. The GPR64 coding region from exon 3 to 8 was replaced by the eGFP-DTA construc. An early stop codon is introduced in intron 8–9. The 3′ homology arm is located in intron 8–9, leading to a partial sequence deletion of GPR64 exons 3–8 in the BAC construct. Multimedia component 1

Multimedia component 2 Figure S2. **Immunhistochemical characterization of *Gpr64***^***mCherry***^**expressing taste bud cells** Representative fluorescence microscopy images of vallate papillae taste bud sections from *Gpr64*^*mCherry*^ immunostained for α-gustducin and double transgenic *Trpm5*^*GFP*^*:Gpr64*^*mCherry*^ reporter mouse with both *Gpr64* promoter-driven mCherry and *Trpm5* promoter-driven GFP expression. Bar = 5 μm Ab, Antibody. Male mice n = 3. Multimedia component 2

Multimedia component 3 Table S1. List of antibodies used for immunofluorescence studies Multimedia component 3

Multimedia component 4 Table S2. List of mRNA-specific probes used for *in situ* hybridization studies Multimedia component 4

Multimedia component 5 Table S3. List of GO-terms used in Figure 6C. Multimedia component 5
