# Supplementary figures and images for: Adhesion receptor ADGRG2/GPR64 is in the GI-tract selectively expressed in mature intestinal tuft cells
Source: Mol Metab. 2021 Apr 5;51:101231. doi: 10.1016/j.molmet.2021.101231 (PMC8105302; doi:10.1016/j.molmet.2021.101231)

Fig. S1.

### Bac GPR64-mCherry

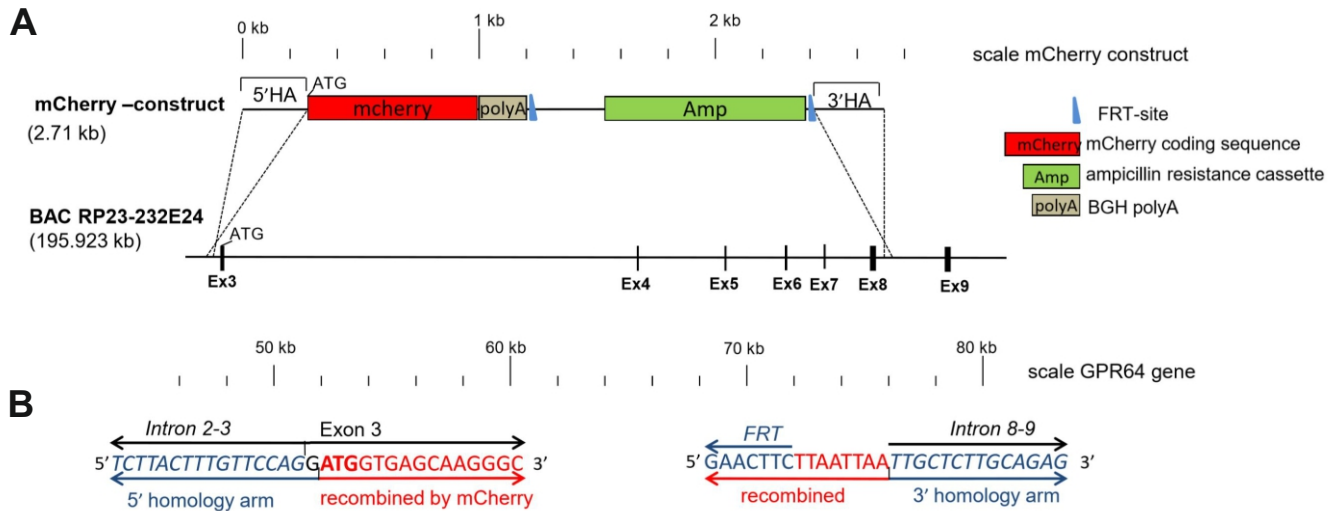

### Bac GPR64-eGFP-DTA

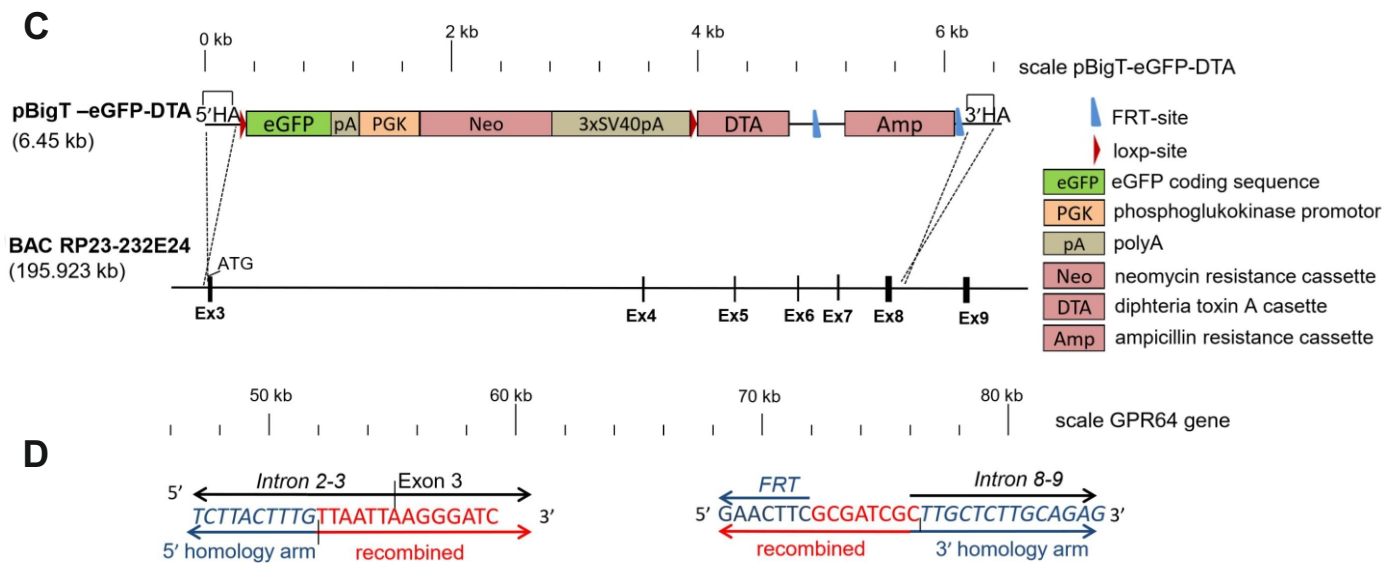

Supplement: Multimedia component 1 — Figure S1. Schematic overview of the generation of the BAC-based Gpr64mCherryand GPR64DTAtransgene A bacterial artificial chromosome (BAC RP23-232E24) carrying the complete GPR64 gene was modified by Red/ET-recombination to insert either a A-B) cassette carrying the mCherry cDNA followed by a polyadenylation signal and an FRT-flanked ampicillin resistance cassette (β-lactamase) to generate the GPR64-mCherry transgene or C-D) a cassette consisting of a floxed eGFP and DTA construct into exon 3 of the GPR64 coding sequence to generate the GPR64-eGFP-DTA transgene. C) The construct contains a splice acceptor donor site, the loxp sequence and the eGFP with a splice acceptor site. The expression of the eGFP is stopped by a polyadenylation site (pA). Directly downstream of the eGFP is a Neomycin expressing cassette (Neo) driven by a phosphoglucokinase (PGK) promoter end stopped by a triple SV40 plolyadenylation site (3xSV40pA). The eGFP /Neo construct is flanked by the two loxp-sites for Cre recombination. Downstream of the second loxp site is the diphtheria toxin A cassette (DTA) located and terminated ba a bovine growth hormone polyadenylation site (polyA). For selection of the recombined BAC in bacteria a FRT-flanked ampicillin resistance cassette (Amp) is located downstream of the DTAn casette. This construct is flanked by a 5 prime and 3 prime homology arm (HA) to allow Red/ET recombination. The partial endogenous locus of the GPR64 gene ranging from exon 3 to 9 is shown below. The regions of recombination are indicated. D) Detailed sequence showing the recombination sites for the 3′ and 5′ homology arms. The GPR64 coding region from exon 3 to 8 was replaced by the eGFP-DTA construc. An early stop codon is introduced in intron 8–9. The 3′ homology arm is located in intron 8–9, leading to a partial sequence deletion of GPR64 exons 3–8 in the BAC construct. [file mmc1.pdf]

Fig. S2.

**Circumvallate papillae - taste bud**

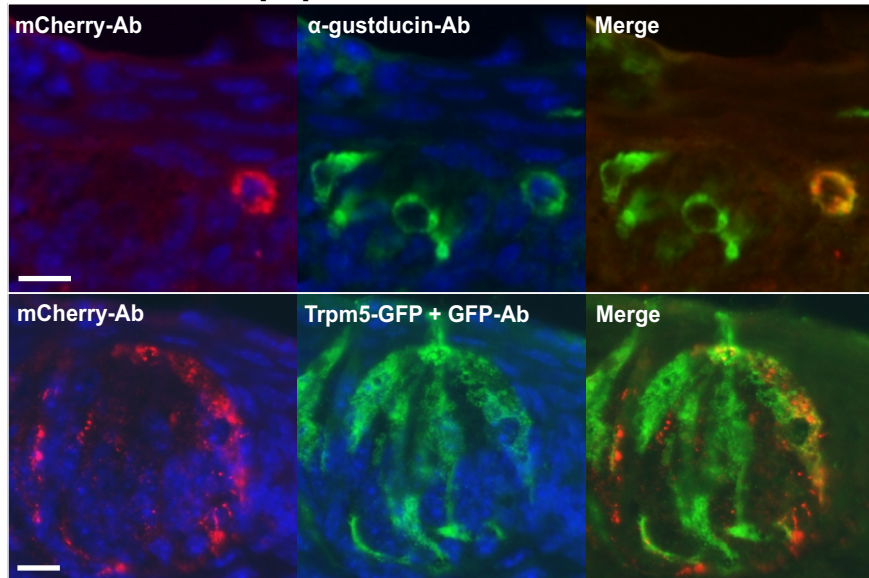

Supplement: Multimedia component 2 — Figure S2. Immunhistochemical characterization of Gpr64mCherryexpressing taste bud cells Representative fluorescence microscopy images of vallate papillae taste bud sections from Gpr64mCherry immunostained for α-gustducin and double transgenic Trpm5GFP:Gpr64mCherry reporter mouse with both Gpr64 promoter-driven mCherry and Trpm5 promoter-driven GFP expression. Bar = 5 μm Ab, Antibody. Male mice n = 3. [file mmc2.pdf]
