# Supplementary material for: Adhesion receptor ADGRG2/GPR64 is in the GI-tract selectively expressed in mature intestinal tuft cells
Source: Mol Metab. 2021 Apr 5;51:101231. doi: 10.1016/j.molmet.2021.101231 (PMC8105302; doi:10.1016/j.molmet.2021.101231)
Supplement: Multimedia component 3 — Table S1. List of antibodies used for immunofluorescence studies [file mmc3.pdf]

Table S1

Peptide/protein target	Manufacturer/Provider, Catalog number	Species	Dilution
Acetylated alpha-tubulin	Sigma Aldrich, T7451	Mouse	1:50 (a.r.)
α-gustducin	Santa Cruz, sc-395	Rabbit	1:100 (a.r.)
Chromogranin A	Immunostar, 20085	Rabbit	1:400
CCK	In house, 8007	Rabbit	1:13000
Cytokeration 18 (CK18)	Progen, 61028	Mouse	1:10 (a.r.)
Doublecortin like kinase 1 (DCLK1)	Abcam, ab37994	Rabbit	1:50
Doublecortin like kinase 1 (DCLK1)	Abcam, ab31704	Rabbit	1:1000
GFP	Abcam, ab6673	Goat	1:1200
GIP	In house, 80867-4	Rabbit	1:6000
Hematopoietic Prostaglandin D synthase (HPGDS)	Cayman Chemical, 160013	Rabbit	1:100 (a.r.)
Secretin	Fahrenkrug, 5585-3	Rabbit	1:3000
Serotonin	Abcam, ab66047	Goat	1:3200
Somatostatin	Santa Cruz, sc-7819	Goat	1:1600
Substance P	In house, 250-2	Rabbit	1:3200
Protein gene product 9.5 (PGP 9.5)	Abcam, ab7291	Mouse	1:4000
Prostaglandin-endoperoxide synthase 1 (PTGS1)	Santa Cruz, sc-1754	Goat	1:50 (a.r.)
Prostaglandin-endoperoxide synthase 2 (PTGS2)	Santa Cruz, sc-1747	Goat	1:500 (a.r.)
PYY	Acris, EUD5201	Guinea pig	1:1000
mCherry	Rockland, 600-401-379	Rabbit	1:400
mCherry	Chromotek, 3F5	Mouse	1:800
AF 488 anti-mouse IgG	Life technologies, A21202	Donkey	1:200
AF 488 anti-rabbit IgG	Life technologies, A21206	Donkey	1:200
AF 568 anti-mouse IgG	Life technologies, A10037	Donkey	1:200
AF 568 anti-rabbit IgG	Life technologies, A10042	Donkey	1:200
AF 488 anti-goat IgG	Life technologies, A10057	Donkey	1:200
CF488 anti-guinea pig IgG	Biotium, 20169	Donkey	1:200

(a.r.): antigen retrieval
