# Supplementary material for: Adhesion receptor ADGRG2/GPR64 is in the GI-tract selectively expressed in mature intestinal tuft cells
Source: Mol Metab. 2021 Apr 5;51:101231. doi: 10.1016/j.molmet.2021.101231 (PMC8105302; doi:10.1016/j.molmet.2021.101231)
Supplement: Multimedia component 4 — Table S2. List of mRNA-specific probes used for in situ hybridization studies [file mmc4.pdf]

Table S2

Target mRNA	Target species	Manufacturer/ Provider and cat. no.
Peptidylprolyl isomerase B (Ppib)	Mus musculus	313911
Gpr64	Mus musculus	430761
Gpr91/Sucnr1	Mus musculus	446981
Glp1r	Mus musculus	447051
Drd3	Mus musculus	447721
Gprc5c	Mus musculus	446991
Gpr41/Ffar3	Mus musculus	447011
Dihydrodipicolinate reductase (DapB)	Bacillus subtilis strain SMY	ACD biotechne, 310043
