# Supplementary material for: Adhesion receptor ADGRG2/GPR64 is in the GI-tract selectively expressed in mature intestinal tuft cells
Source: Mol Metab. 2021 Apr 5;51:101231. doi: 10.1016/j.molmet.2021.101231 (PMC8105302; doi:10.1016/j.molmet.2021.101231)
Supplement: Multimedia component 5 — Table S3. List of GO-terms used in Figure 6C. [file mmc5.pdf]

signif_rank	LFC_weightedmean	LFC_precision	genesetoverlap	minlogpval	GO.ID	GO.term
1	-2,835109585	2,107972002	427	28,89819573	GO:0007005	mitochondrion organization
2	-8,730575066	2,603039657	255	25,93872994	GO:0042254	ribosome biogenesis
3	-7,070284369	2,487619425	394	24,24729125	GO:0022613	ribonucleoprotein complex biogenesis
4	-8,739436736	2,588412092	207	22,4845338	GO:0016072	rRNA metabolic process
5	-2,626894406	1,927771074	254	21,53919789	GO:0009123	nucleoside monophosphate metabolic process
6	0,485894438	1,946877651	451	19,9386729	GO:0051186	cofactor metabolic process
7	-2,830100758	1,970063855	240	19,73152315	GO:0009167	purine ribonucleoside monophosphate metabolic process
8	-2,759711073	1,817583128	264	19,59653064	GO:0009141	nucleoside triphosphate metabolic process
9	-6,312429381	2,739893154	325	19,57057592	GO:0034470	ncRNA processing
10	-9,43358458	5,821166728	71	19,28701229	GO:0033108	mitochondrial respiratory chain complex assembly
11	-14,66732433	3,731780367	59	18,21492109	GO:0042255	ribosome assembly
12	-0,439738361	1,821001383	405	17,50554233	GO:0019693	ribose phosphate metabolic process
13	-5,209594407	2,766344395	434	17,17784408	GO:0034660	ncRNA metabolic process
14	-0,402496441	1,799617403	379	16,64585246	GO:0009150	purine ribonucleotide metabolic process
15	-7,476019302	2,411801084	88	16,37688618	GO:0006119	oxidative phosphorylation
16	-2,123907785	2,211354598	331	16,2448795	GO:0006091	generation of precursor metabolites and energy
17	-11,89765195	7,550355814	42	15,84704371	GO:0010257	NADH dehydrogenase complex assembly
18	-11,89765195	7,550355814	42	15,84704371	GO:0032981	mitochondrial respiratory chain complex I assembly
19	0,454274249	1,754652412	441	14,89690327	GO:0072521	purine-containing compound metabolic process
20	-12,85309382	3,563870175	67	13,93055518	GO:0042273	ribosomal large subunit biogenesis
21	-2,415159652	2,002044224	162	13,83499427	GO:0006839	mitochondrial transport
22	-2,732094932	1,995266576	150	13,59540768	GO:0045333	cellular respiration
23	-6,843679077	2,495313973	218	13,57545771	GO:0071826	ribonucleoprotein complex subunit organization
24	-13,32305488	2,114318205	70	13,20997491	GO:0002181	cytoplasmic translation
25	-5,181882963	2,296707418	85	13,07270365	GO:0022900	electron transport chain
26	-2,835510682	1,753927463	120	12,66939946	GO:0009206	purine ribonucleoside triphosphate biosynthetic process
27	-7,917587852	2,328693551	64	12,38509326	GO:1990542	mitochondrial transmembrane transport
28	-2,713694597	1,824793322	134	12,28066999	GO:0009142	nucleoside triphosphate biosynthetic process
29	-11,78031611	3,058188232	59	12,16213939	GO:0042274	ribosomal small subunit biogenesis
30	-4,934478628	2,826831157	339	11,95785412	GO:0008380	RNA splicing
31	-5,314879933	2,207002564	79	11,89542481	GO:0022904	respiratory electron transport chain
32	-7,577596827	2,967594626	57	11,83934499	GO:0042775	mitochondrial ATP synthesis coupled electron transport
33	-6,216865837	2,528468599	61	10,86428809	GO:0042773	ATP synthesis coupled electron transport
34	-1,48096719	1,878211285	143	10,6912067	GO:0009124	nucleoside monophosphate biosynthetic process
35	-9,692948725	2,444610153	72	10,53565869	GO:0032543	mitochondrial translation
36	-0,286294323	1,971795612	219	10,10425553	GO:0051188	cofactor biosynthetic process
37	-6,714700412	2,434056876	87	10,05615516	GO:0070585	protein localization to mitochondrion
38	-1,690124947	2,213472305	234	9,97706876	GO:0015980	energy derivation by oxidation of organic compounds
39	-1,338946912	1,882806892	136	9,865538446	GO:0009156	ribonucleoside monophosphate biosynthetic process
40	-0,075564922	1,801643921	206	9,626324013	GO:0046390	ribose phosphate biosynthetic process
41	-23,39923911	15,2341023	17	9,290695868	GO:0000028	ribosomal small subunit assembly
42	-4,710800864	2,526348571	93	9,146882185	GO:0007006	mitochondrial membrane organization
43	-0,306272897	1,773049042	191	9,103942745	GO:0009152	purine ribonucleotide biosynthetic process
44	0,253763835	1,933774476	259	8,885297226	GO:1901293	nucleoside phosphate biosynthetic process
45	1,410559201	1,966195612	294	8,758465215	GO:0006732	coenzyme metabolic process
46	-13,25732942	4,677895976	29	8,736906789	GO:0000027	ribosomal large subunit assembly
47	-1,288033422	1,58186122	144	8,20864267	GO:0034404	nucleobase-containing small molecule biosynthetic process
48	-13,84709384	4,487273791	22	8,068709316	GO:0010499	proteasomal ubiquitin-independent protein catabolic process

49	-0,046392961	1,793113159	213	8,049955544	GO:0072522	purine-containing compound biosynthetic process
50	-4,146705083	1,853883991	71	7,991335869	GO:1903578	regulation of ATP metabolic process
51	-3,719586857	2,690913872	418	7,842991721	GO:0006397	mRNA processing
52	-11,04676475	3,420038857	38	7,089148137	GO:0044743	protein transmembrane import into intracellular organelle
53	-2,809286165	1,902157758	88	7,03502359	GO:0006140	regulation of nucleotide metabolic process
54	-2,758726783	2,007194702	102	6,867145333	GO:0009132	nucleoside diphosphate metabolic process
55	0,069171407	2,411172568	168	6,715083348	GO:0009108	coenzyme biosynthetic process
56	-3,770414887	2,175301095	49	6,564865852	GO:0006749	glutathione metabolic process
57	-14,62237544	4,400734713	21	6,563028666	GO:0000469	cleavage involved in rRNA processing
58	1,605858422	1,876788818	151	6,437328624	GO:0006733	oxidoreduction coenzyme metabolic process
59	-14,07507587	2,527924347	29	6,28519729	GO:0000387	spliceosomal snRNP assembly
60	8,539547358	2,578739923	92	6,237980106	GO:0006892	post-Golgi vesicle-mediated transport
61	-2,126803392	2,055543347	82	6,188636614	GO:0009179	purine ribonucleoside diphosphate metabolic process
62	-9,204455527	2,450373295	57	6,187691895	GO:0019080	viral gene expression
63	-7,687680724	2,63922132	54	5,915508727	GO:0071806	protein transmembrane transport
64	-2,543016739	1,92549202	86	5,903258971	GO:0046939	nucleotide phosphorylation
65	-0,684410231	1,88250033	420	5,893393506	GO:0019439	aromatic compound catabolic process
66	-7,832208118	2,597962662	53	5,880350158	GO:0065002	intracellular protein transmembrane transport
67	-3,317331635	1,901156939	101	5,872573165	GO:0043467	regulation of generation of precursor metabolites and energy
68	-0,34925009	1,650533627	111	5,842196432	GO:0009166	nucleotide catabolic process
69	8,687369015	1,541581713	324	5,833478674	GO:0002443	leukocyte mediated immunity
70	8,557687158	1,548696134	209	5,823178711	GO:0002703	regulation of leukocyte mediated immunity
71	-0,289983512	1,876670333	444	5,783830981	GO:1901361	organic cyclic compound catabolic process
72	-7,019031481	2,509630753	28	5,735283608	GO:0007007	inner mitochondrial membrane organization
73	-8,88411311	2,819037119	40	5,719522124	GO:0030490	maturation of SSU-rRNA
74	0,305568197	1,602129707	121	5,572103707	GO:1901292	nucleoside phosphate catabolic process
75	1,200303199	2,017291696	250	5,457297988	GO:0006790	sulfur compound metabolic process
76	-1,781067104	2,251161284	107	5,433773798	GO:0008637	apoptotic mitochondrial changes
77	-0,451924174	1,844833225	406	5,405131924	GO:0046700	heterocycle catabolic process
78	-1,253826397	1,909264125	366	5,281668752	GO:0034655	nucleobase-containing compound catabolic process
79	2,76299229	1,865034777	447	5,27811487	GO:0090407	organophosphate biosynthetic process
80	-1,376648283	1,994539335	49	5,264997478	GO:0010823	negative regulation of mitochondrion organization
81	5,941479365	1,543124832	44	5,236550366	GO:0033003	regulation of mast cell activation
82	-0,712653574	1,867261937	400	5,222766244	GO:0044270	cellular nitrogen compound catabolic process
83	-6,265529266	1,455255506	33	5,219009504	GO:0006220	pyrimidine nucleotide metabolic process
84	-8,988151025	1,641552801	20	5,177998338	GO:0006783	heme biosynthetic process
85	1,805363945	1,761296412	133	5,0397885	GO:0046496	nicotinamide nucleotide metabolic process
86	-3,301193196	2,682747698	140	4,993219539	GO:0006457	protein folding
87	-2,33541661	2,549746425	186	4,99120789	GO:0071824	protein-DNA complex subunit organization
88	-1,310103139	2,801145102	255	4,988485368	GO:0006520	cellular amino acid metabolic process
89	6,936616141	2,289244917	253	4,777825915	GO:0048193	Golgi vesicle transport
90	-3,800105122	1,910565012	57	4,749428434	GO:0001836	release of cytochrome c from mitochondria
91	-3,943644336	2,960354094	161	4,717257798	GO:0006399	tRNA metabolic process
92	1,74874613	1,83043019	139	4,715616291	GO:0072524	pyridine-containing compound metabolic process
93	-12,51886674	4,826315354	14	4,562034346	GO:0034471	ncRNA 5'-end processing
94	4,954730573	2,028978403	201	4,542500809	GO:0006898	receptor-mediated endocytosis
95	7,774945946	1,688319154	245	4,524395357	GO:0051056	regulation of small GTPase mediated signal transduction
96	7,907722054	2,301889923	121	4,475515048	GO:0007030	Golgi organization
97	-5,070974728	2,210465088	28	4,436480149	GO:0017004	cytochrome complex assembly

98	-8,777325978	1,776001576	25	4,424995849	GO:0006221	pyrimidine nucleotide biosynthetic process
99	-25,42877966	2,808889574	14	4,297029595	GO:0019081	viral translation
100	0,927358588	1,767420575	124	4,270757493	GO:0010821	regulation of mitochondrion organization
101	-7,144431758	5,232360016	19	4,266951477	GO:0031163	metallo-sulfur cluster assembly
102	-7,144431758	5,232360016	19	4,266951477	GO:0016226	iron-sulfur cluster assembly
103	1,630021773	2,424085268	295	4,262406934	GO:0044282	small molecule catabolic process
104	-0,653877666	1,374919433	39	4,252946487	GO:0098754	detoxification
105	1,946485543	1,847179953	266	4,245836838	GO:0051604	protein maturation
106	-1,147344261	2,064501448	73	4,224562456	GO:0042866	pyruvate biosynthetic process
107	16,3740074	0,895160214	62	4,183788144	GO:0045576	mast cell activation
108	-27,32983112	4,763714204	10	4,1787537	GO:0075522	IRES-dependent viral translational initiation
109	7,592788244	1,52618859	146	4,14151797	GO:0002705	positive regulation of leukocyte mediated immunity
110	-7,201758793	1,763904418	29	4,1272343	GO:0042168	heme metabolic process
111	6,11384644	1,690822981	461	4,056883896	GO:0007264	small GTPase mediated signal transduction
112	-3,885750298	2,894476929	111	4,029334911	GO:0008033	tRNA processing
113	-0,916291042	2,564762874	188	4,011924104	GO:1901605	alpha-amino acid metabolic process
114	-11,48564651	4,634703381	13	4,004644491	GO:0099116	tRNA 5'-end processing
115	6,35397828	2,008139781	110	3,993600061	GO:0031623	receptor internalization
116	18,30849279	2,325802547	23	3,983622832	GO:0046856	phosphatidylinositol dephosphorylation
117	0,409616087	2,138220819	83	3,955582758	GO:0019359	nicotinamide nucleotide biosynthetic process
118	-11,92236767	1,607004696	16	3,953195689	GO:0009147	pyrimidine nucleoside triphosphate metabolic process
119	6,541932054	2,300034417	194	3,952153106	GO:0034329	cell junction assembly
120	1,13444558	1,57797249	86	3,935673218	GO:1902600	proton transmembrane transport
121	-1,630946946	2,440100095	151	3,919488533	GO:0065004	protein-DNA complex assembly
122	-7,363470484	1,792147594	26	3,862933011	GO:0019692	deoxyribose phosphate metabolic process
123	-7,363470484	1,792147594	26	3,862933011	GO:0009394	2'-deoxyribonucleotide metabolic process
124	0,539479185	2,162837112	96	3,85537035	GO:0006090	pyruvate metabolic process
125	-3,013205988	2,290250721	297	3,854422427	GO:0006417	regulation of translation
126	2,88546761	2,109874444	383	3,838573741	GO:0001667	ameboidal-type cell migration
127	5,084787941	1,773076612	54	3,819951919	GO:0002886	regulation of myeloid leukocyte mediated immunity
128	-4,789710219	1,520603806	14	3,803053982	GO:0009084	glutamine family amino acid biosynthetic process
129	-8,838391964	2,169051773	44	3,799889155	GO:0006414	translational elongation
130	-5,440623233	2,354146711	20	3,778571351	GO:0033599	regulation of mammary gland epithelial cell proliferation
131	-10,46084884	4,956367666	11	3,735337973	GO:0001682	tRNA 5'-leader removal
132	-19,85598108	5,377012331	9	3,71942001	GO:0006183	GTP biosynthetic process
133	6,584045204	1,679868048	265	3,715112706	GO:0002460	adaptive immune response based on somatic recombination of immune receptors built from immunoglobulin superfamily domains
134	8,047485154	1,600055977	214	3,6485723	GO:0046578	regulation of Ras protein signal transduction
135	-2,427348401	1,954349761	111	3,638537991	GO:1902806	regulation of cell cycle G1/S phase transition
136	8,255306412	2,038226443	101	3,63106406	GO:0006939	smooth muscle contraction
137	0,888898165	1,888108444	352	3,623388737	GO:0072594	establishment of protein localization to organelle
138	-1,633181713	2,472229601	50	3,614848789	GO:0046902	regulation of mitochondrial membrane permeability
139	6,922775109	1,739953407	117	3,607021324	GO:0016999	antibiotic metabolic process
140	1,759167634	2,099331914	39	3,598003651	GO:0045910	negative regulation of DNA recombination
141	17,86872557	1,816409152	21	3,59376383	GO:0031649	heat generation
142	0,415791604	2,273023129	90	3,590995057	GO:0072525	pyridine-containing compound biosynthetic process
143	12,20277565	4,113294296	11	3,561145166	GO:0045717	negative regulation of fatty acid biosynthetic process
144	1,101046707	1,987853334	327	3,560147845	GO:0016053	organic acid biosynthetic process
145	2,679765799	2,323443548	100	3,544350181	GO:0048259	regulation of receptor-mediated endocytosis
146	10,25615759	1,659667402	24	3,544191042	GO:0045332	phospholipid translocation

147	-4,165342528	13,59221876	16	3,537324512	GO:1901663	quinone biosynthetic process
148	8,938834393	1,797546155	51	3,506245711	GO:0050775	positive regulation of dendrite morphogenesis
149	1,320859112	2,308888817	32	3,491851649	GO:1903146	regulation of autophagy of mitochondrion
150	-13,32158727	2,971659764	14	3,474795353	GO:0090151	establishment of protein localization to mitochondrial membrane
151	2,914731584	2,469414529	125	3,448814827	GO:0048675	axon extension
152	-0,500630355	2,088452898	358	3,423215176	GO:0043161	proteasome-mediated ubiquitin-dependent protein catabolic process
153	-0,537246272	2,197726783	420	3,411719817	GO:0010498	proteasomal protein catabolic process
154	1,16577292	1,995160174	326	3,383801266	GO:0046394	carboxylic acid biosynthetic process
155	2,098214043	2,30878553	44	3,381835257	GO:0030517	negative regulation of axon extension
156	-3,12425483	3,135588625	130	3,368026107	GO:0009451	RNA modification
157	-12,40323498	2,972901229	17	3,36657825	GO:1904666	regulation of ubiquitin protein ligase activity
158	2,519139663	2,268994364	237	3,359152674	GO:0048588	developmental cell growth
159	4,311689248	1,992012679	215	3,35433649	GO:0017157	regulation of exocytosis
160	-3,074927604	2,186704389	116	3,350302102	GO:0006338	chromatin remodeling
161	6,039285999	1,912061607	183	3,346609103	GO:0043112	receptor metabolic process
162	1,996693149	2,489167827	207	3,336040229	GO:0046395	carboxylic acid catabolic process
163	-11,5592829	3,134536067	21	3,327328464	GO:0000470	maturation of LSU-rRNA
164	-0,193405586	2,065377476	269	3,32466041	GO:0009895	negative regulation of catabolic process
165	6,343560049	2,481274192	81	3,320474115	GO:0008589	regulation of smoothened signaling pathway
166	-1,639016735	2,213260936	404	3,318762913	GO:0010608	posttranscriptional regulation of gene expression
167	5,407526806	3,184027846	34	3,317069009	GO:0045601	regulation of endothelial cell differentiation
168	-1,325364827	1,99284757	143	3,308399067	GO:2000058	regulation of ubiquitin-dependent protein catabolic process
169	0,681794703	1,394209883	44	3,3052335	GO:0042743	hydrogen peroxide metabolic process
170	-0,868932503	2,022629988	63	3,292403158	GO:0006081	cellular aldehyde metabolic process
171	-8,005346418	3,451169125	27	3,285959096	GO:0000460	maturation of 5.8S rRNA
172	-0,479809345	2,163356506	117	3,269760867	GO:0016052	carbohydrate catabolic process
173	-15,70392122	2,733751478	12	3,246538633	GO:1901070	guanosine-containing compound biosynthetic process
174	-13,92004564	2,909718264	14	3,235914409	GO:0043248	proteasome assembly
175	5,196418144	2,992118686	84	3,228534975	GO:0007041	lysosomal transport
176	-1,323347958	2,12261432	330	3,216305348	GO:0045787	positive regulation of cell cycle
177	-1,333538668	2,149316464	236	3,206971459	GO:0051348	negative regulation of transferase activity
178	9,511273383	1,276123832	183	3,202545421	GO:0002274	myeloid leukocyte activation
179	7,039068999	1,789988006	240	3,195740577	GO:0034330	cell junction organization
180	1,133348473	3,013719523	84	3,194842501	GO:1903008	organelle disassembly
181	-4,997421388	1,943206247	12	3,189806926	GO:0051198	negative regulation of coenzyme metabolic process
182	-4,997421388	1,943206247	12	3,189806926	GO:0030812	negative regulation of nucleotide catabolic process
183	-11,0810523	4,719026131	11	3,187662578	GO:0000491	small nucleolar ribonucleoprotein complex assembly
184	4,949655709	1,857773942	42	3,178429605	GO:0043300	regulation of leukocyte degranulation
185	-6,69522083	1,469836291	26	3,161856979	GO:0033014	tetrapyrrole biosynthetic process
186	0,348106538	2,01632182	63	3,14222077	GO:0009064	glutamine family amino acid metabolic process
187	-6,636726973	1,637530445	30	3,126621612	GO:1901659	glycosyl compound biosynthetic process
188	5,158555739	1,772234248	86	3,120995012	GO:0042310	vasoconstriction
189	13,38590031	1,137844586	62	3,116106428	GO:0043299	leukocyte degranulation
190	-4,188908209	2,351036496	108	3,114191295	GO:0045727	positive regulation of translation
191	-11,08823807	1,695255511	11	3,111876158	GO:0009264	deoxyribonucleotide catabolic process
192	2,302420009	2,273641846	99	3,090484245	GO:0030048	actin filament-based movement
193	11,70509754	1,204412182	86	3,090337704	GO:0002444	myeloid leukocyte mediated immunity
194	-13,48348469	4,42910334	9	3,090228012	GO:0000492	box C/D snoRNP assembly
195	5,191920341	1,791746234	345	3,082772354	GO:0006887	exocytosis

196	-5,139258205	2,438001376	35	3,0601216	GO:0018208	peptidyl-proline modification
197	-1,299224982	1,724515588	36	3,0546995	GO:0030811	regulation of nucleotide catabolic process
198	-5,454033334	2,356464226	18	3,046315637	GO:0008535	respiratory chain complex IV assembly
199	-8,539424799	3,362765544	24	3,037901282	GO:0070199	establishment of protein localization to chromosome
200	-4,03539373	1,776539162	19	3,032533996	GO:0071157	negative regulation of cell cycle arrest
201	-2,209576272	2,329947721	340	3,030303034	GO:0034248	regulation of cellular amide metabolic process
202	7,740649391	2,137732248	90	3,02750703	GO:0099072	regulation of postsynaptic membrane neurotransmitter receptor levels
203	4,433648163	0,951222956	10	3,006279702	GO:0043497	regulation of protein heterodimerization activity
204	0,148593065	1,724784925	61	2,989882812	GO:0051193	regulation of cofactor metabolic process
205	-4,629960713	2,175260907	47	2,988233439	GO:2000059	negative regulation of ubiquitin-dependent protein catabolic process
206	9,984293659	3,202488122	22	2,972607647	GO:0072337	modified amino acid transport
207	-2,865956886	2,872992579	39	2,946892312	GO:0006284	base-excision repair
208	-0,063853656	2,022893454	224	2,944501511	GO:0031330	negative regulation of cellular catabolic process
209	-11,28273578	1,783776245	13	2,944327116	GO:0009148	pyrimidine nucleoside triphosphate biosynthetic process
210	-6,512016056	2,166206506	56	2,937125954	GO:0090501	RNA phosphodiester bond hydrolysis
211	-12,16407489	1,543234839	10	2,935087443	GO:0046386	deoxyribose phosphate catabolic process
212	3,151412859	2,076689381	267	2,91753147	GO:0090130	tissue migration
213	7,111709487	1,320436724	358	2,906835	GO:0002697	regulation of immune effector process
214	-0,002894428	1,814428786	373	2,906619519	GO:0044419	interspecies interaction between organisms
215	-7,634943472	1,4165714	26	2,903269554	GO:0042455	ribonucleoside biosynthetic process
216	-2,326368366	2,678463966	126	2,898801824	GO:0006333	chromatin assembly or disassembly
217	-10,42917711	2,412062883	16	2,892580143	GO:0000966	RNA 5'-end processing
218	-0,34550704	4,712272452	13	2,891753479	GO:0009226	nucleotide-sugar biosynthetic process
219	-4,118253101	2,254110231	104	2,871015794	GO:0006413	translational initiation
220	8,19516429	2,559795861	56	2,869234975	GO:0006893	Golgi to plasma membrane transport
221	3,322908185	2,894424069	56	2,861646428	GO:0031122	cytoplasmic microtubule organization
222	4,687656883	1,77274868	120	2,847109192	GO:0035637	multicellular organismal signaling
223	-3,314195988	2,657043985	36	2,846375731	GO:0045684	positive regulation of epidermis development
224	11,41775656	1,156080229	63	2,844798306	GO:0051057	positive regulation of small GTPase mediated signal transduction
225	3,172352325	2,064879129	265	2,837858136	GO:0010631	epithelial cell migration
226	-2,321617678	2,144357376	82	2,830162722	GO:0051289	protein homotetramerization
227	-5,133354106	2,085443883	12	2,828858361	GO:0019646	aerobic electron transport chain
228	6,964359597	1,261875882	225	2,826711917	GO:0002263	cell activation involved in immune response
229	2,21900487	2,265165308	408	2,826602174	GO:0007409	axonogenesis
230	0,687558359	2,77859933	62	2,82030527	GO:0061726	mitochondrion disassembly
231	-8,605145238	3,591473514	10	2,816597778	GO:0072321	chaperone-mediated protein transport
232	-12,25577937	1,681497375	14	2,805937065	GO:0046132	pyrimidine ribonucleoside biosynthetic process
233	-3,922002074	3,818923056	15	2,799609398	GO:0042407	cristae formation
234	14,46880453	1,231116877	11	2,787081903	GO:0033004	negative regulation of mast cell activation
235	-1,807658035	3,023683205	72	2,784534498	GO:2000573	positive regulation of DNA biosynthetic process
236	2,336024105	2,205803746	92	2,763928196	GO:0043648	dicarboxylic acid metabolic process
237	2,9213822	2,064893471	78	2,762791875	GO:0070252	actin-mediated cell contraction
238	11,41005917	1,982088816	45	2,760785342	GO:0019369	arachidonic acid metabolic process
239	4,966456574	1,710653734	426	2,75308403	GO:0055074	calcium ion homeostasis
240	-1,175644855	2,447029354	59	2,751920064	GO:0090559	regulation of membrane permeability
241	-2,927390184	2,216541743	57	2,746764103	GO:1901607	alpha-amino acid biosynthetic process
242	0,049401391	1,181977642	50	2,746727875	GO:0072527	pyrimidine-containing compound metabolic process
243	-2,382718209	2,657899675	12	2,742094395	GO:0060433	bronchus development
244	4,012871171	1,978770788	176	2,741430837	GO:0048771	tissue remodeling

245	-3,370236321	2,067186022	56	2,738968106	GO:0071156	regulation of cell cycle arrest
246	10,48503879	1,424179323	41	2,73057951	GO:0001961	positive regulation of cytokine-mediated signaling pathway
247	4,028165959	2,305725565	233	2,711533614	GO:0099003	vesicle-mediated transport in synapse
248	3,637568311	2,009685264	151	2,709570598	GO:0035150	regulation of tube size
249	3,179346865	2,482613207	53	2,700348322	GO:0098661	inorganic anion transmembrane transport
250	5,581409642	2,295396497	30	2,688960098	GO:0098884	postsynaptic neurotransmitter receptor internalization
